# Past wounds, present bonds, future mothers: item-level analysis of perinatal risk profiles

**DOI:** 10.3389/fpsyg.2026.1899047

**Published:** 2026-10-09

**Authors:** Raffaella Mineo, Leonardo Fazio, Antonio Maiorana, Claudia Guarneri, Jada Sottile, Eleonora Bevacqua, Linda A. Antonucci, Maria Rita Infurna

**Affiliations:** 1 Department of Psychology, Educational Science and Human Movement, University of Palermo, Palermo, Italy; 2 Department of Medicine and Surgery, LUM University “Giuseppe Degennaro”, Bari, Italy; 3 HCU Obstetrics and Gynecology, ARNAS Civico Di Cristina-Benfratelli Hospital, Palermo, Italy; 4 Department of Translational Biomedicine and Neuroscience, University of Bari Aldo Moro, Bari, Italy

**Keywords:** childhood trauma, adverse childhood experiences, cluster analysis, dyadic adjustment, maternal antenatal attachment, perinatal psychological distress

## Abstract

**Background:**

Perinatal depression is shaped by interacting psycho-relational risk factors—childhood adversity, current relational functioning, perceived social support, and prenatal attachment. Variable-centered approaches focused on isolated predictors may obscure how these dimensions combine within individuals to generate distinct vulnerability configurations.

**Objective:**

To identify person-centered profiles of psychosocial functioning during pregnancy and to examine their association with depressive symptoms across the perinatal period.

**Participants and setting:**

A sample of 774 pregnant women was recruited from full-term and high-risk pregnancy clinics at one of the largest birth centers in Southern Italy. A subsample of 200 women (25.8%) completed the postpartum follow-up at 6 months (T1).

**Methods:**

An item-level k-means cluster analysis (*k* = 3) was performed on 44 items from the CTQ-SF, DAS-7, MSSS, and MAAS. Cluster differences in EPDS scores were tested cross-sectionally with one-way ANOVA and a covariate-adjusted model (T0), and longitudinally with a repeated-measures ANOVA (T0–T1). A formal attrition analysis and domain-level sensitivity analyses were also conducted.

**Results:**

Three profiles emerged: Low-Risk (53.7%), Moderately Adjusted (31.7%), and High-Adversity (14.6%). Between-cluster differences in depressive symptoms were significant at T0 (*F*(2, 771) = 62.52, *p* < 0.001, *η*^2^ = 0.140), were essentially unchanged after adjustment for sociodemographic and obstetric covariates (*F*(2, 747) = 58.15, *p* < 0.001, partial *η*^2^ = 0.135), and persisted across the perinatal period (main effect of cluster: *F*(2, 197) = 22.74, *p* < 0.001, partial *η*^2^ = 0.188), with no significant Time effect (*F*(1, 197) = 0.38, *p* = 0.54) or Cluster × Time interaction (*F*(2, 197) = 1.40, *p* = 0.25). At T0, 11.1, 35.9, and 44.2% of the Low-Risk, Moderately Adjusted, and High-Adversity profiles, respectively, scored above the EPDS clinical cut-off (≥ 9; *p* < 0.001).

**Conclusion:**

Item-level cluster analysis reveals clinically significant heterogeneity in perinatal risk. Between-profile differences in depressive symptoms persisted across the perinatal period and may inform stratified, trauma-informed prevention strategies.

## 1 Introduction

Perinatal depression is a multifactorial condition whose etiology extends well beyond hormonal fluctuations to encompass a range of psycho-relational risk factors that emerge and interact during pregnancy (Longoria et al., 2024). Early life experiences, especially childhood abuse and neglect, make pregnant women more susceptible to depressive symptoms through multiple pathways (Infurna et al., 2023; Longoria et al., 2024), including mechanisms of emotional reactivation (Shamblaw et al., 2018). Both emotional and physical neglect are significant predictors of depression during and after pregnancy (Li et al., 2017; Tebeka et al., 2021). This association appears to follow a dose–response pattern, with cumulative exposure to different forms of childhood trauma conferring progressively greater risk (Shamblaw et al., 2018). Meta-analytic evidence has consolidated this link: adverse childhood experiences show significant pooled associations with both maternal depression and maternal anxiety across the perinatal period (Racine et al., 2021), and cohort data indicate that childhood adversity remains a robust predictor of depressive symptoms in pregnancy even when concurrent psychosocial stressors are taken into account (Wajid et al., 2020). Early adversity can therefore be conceptualized as a distal vulnerability factor whose effects are carried into the reproductive years and re-emerge at developmentally sensitive transitions such as pregnancy, when caregiving representations are reactivated.

However, this link is not deterministic. Resilience constitutes the necessary counterpart of risk in this developmental equation: among pregnant women exposed to childhood adversity, higher resilience has been associated with markedly lower odds of prenatal mood, anxiety, and behavioral health conditions (Young-Wolff et al., 2019). Notably, resilience in this context appears to be fundamentally relational: relational resilience—the capacity to establish and mobilize growth-fostering, supportive relationships—has been shown to mediate the pathway from adverse childhood experiences to prenatal depressive symptoms (Howell et al., 2020). The expression of early vulnerability may thus be amplified by current psychosocial risk conditions, including intimate partner violence, low social support, and relationship dissatisfaction (Choi and Sikkema, 2016; Inanici et al., 2017), or attenuated by precisely those relational resources that person-centered approaches allow to be examined jointly with risk.

Current relational functioning appears relevant in shaping maternal emotional adjustment especially during the perinatal transition. Positive couple dynamics, perceived support, and effective dyadic coping are associated with lower depressive risk and greater psychological resilience (Alves et al., 2019; Molgora et al., 2019; Thiel et al., 2020). Conversely, relationships marked by conflict, emotional unavailability, or poor relational attunement may intensify vulnerability, reinforcing insecurity, isolation, and difficulties in emotion regulation (Pilkington et al., 2015; Zhao et al., 2025). The impact of childhood adversity on perinatal mental health is therefore likely to depend not only on the severity of early adverse experiences, but also on the quality of current relational resources.

A further key dimension is prenatal attachment, defined as the mother’s emotional bond with her unborn child. Evidence suggests that weaker antenatal attachment is both associated with and potentially predictive of perinatal depressive symptomatology (Berthelot et al., 2020; Infurna et al., 2024). Depressive symptoms may reduce emotional investment in the fetus, while low prenatal attachment may itself increase susceptibility to distress, potentially generating self-reinforcing cycles of vulnerability (Mangialavori et al., 2024). At the same time, even in contexts marked by adversity, prenatal attachment may represent a meaningful relational resource and a potential opportunity for reorganization during the transition to motherhood (van Vugt and Versteegh, 2020).

Despite these advancements, the literature has predominantly employed variable-centered methodologies, focusing on isolated predictors and linear associations among risk factors. Although these models have provided significant insights, they may be less effective in capturing the complex, dynamic, and potentially non-linear interactions between childhood trauma, relational functioning, perceived social support, and antenatal attachment (Laursen and Hoff, 2006). Women with similar overall scores may exhibit markedly different psychosocial profiles and developmental trajectories. Person-centered approaches, such as cluster analysis, offer a valuable alternative by identifying naturally occurring subgroups of individuals who share similar multivariate configurations across domains, thereby emphasizing within-person patterns rather than between-variable relationships (Bergman and Magnusson, 1997). In doing so, these methods preserve clinically meaningful heterogeneity that aggregate models may obscure, enabling a more refined characterization of psychosocial functioning (Lamers et al., 2010).

This perspective is particularly important in the perinatal context, which is inherently dynamic and developmentally sensitive. Pregnancy is not a static condition, but a period of ongoing emotional, relational, and physiological transformation, during which early vulnerabilities may be reactivated, mitigated, or reorganized in light of current experiences and bonds (McCarthy et al., 2021; van den Bergh et al., 2020). Understanding perinatal risk, therefore, requires approaches capable of identifying not only how much risk is present, but also how different dimensions combine within individuals to shape distinct configurations of vulnerability and protection.

## 2 Materials and methods

### 2.1 Study design

The present study employed an observational, prospective cohort design with two assessment waves, nested within a hospital-based perinatal psychosocial screening program aimed at identifying risk and resilience profiles during pregnancy. Psychosocial functioning and depressive symptoms were assessed during pregnancy (T0), and depressive symptoms were re-assessed at 6 months postpartum (T1). The person-centered analyses were conducted cross-sectionally on the baseline sample, whereas the longitudinal component examined the persistence of between-profile differences in depressive symptomatology across the perinatal period.

The protocol received approval from the Bioethics Committee at the University of Palermo (167/2023, approved 26/10/2023) and the Ethics Committee at “Civico–Di Cristina–Benfratelli” Hospital (142/2022, approved 24/06/2022), conducted according to the Declaration of Helsinki (2024).

### 2.2 Sample size considerations

No *a priori* power analysis was performed: consistent with the screening nature of the parent project, the sample size was determined pragmatically by the number of eligible women consecutively approached during the recruitment window. The adequacy of the achieved sample (*N* = 774) was nonetheless evaluated on three grounds. First, the smallest cluster comprised 113 women, ensuring stable within-profile estimates. Second, the robustness of the clustering solution was formally verified through bootstrap resampling (1,000 iterations; Jaccard coefficients 0.81–0.93, above the conventional ≥ 0.75 threshold for cluster stability; Hennig, 2007) and through two domain-level sensitivity analyses that reproduced the three-profile solution, one with equal weight per domain (adjusted Rand index = 0.76) and one strictly balanced across instruments, with a single standardized score per instrument (adjusted Rand index = 0.73; Supplementary Table S1). Third, a sensitivity power analysis (G*Power 3.1; *α* = 0.05, 1 − *β* = 0.80, three groups) indicated that the baseline sample allowed the detection of between-profile effects on the EPDS as small as *f* ≈ 0.11, and the longitudinal subsample (*n* = 200) of effects of *f* ≈ 0.22.

### 2.3 Recruitment and procedure

Participants were recruited between January 2024 and March 2025 at the full-term and high-risk pregnancy outpatient clinics of the ARNAS “Civico–Di Cristina–Benfratelli” Hospital in Palermo, which hosts one of the highest-volume obstetric units in Southern Italy. During routine antenatal visits, eligible women were consecutively approached in the clinic waiting areas by trained psychologists belonging to the research team and not involved in the women’s clinical care. The study was introduced as a university research initiative complementary to, but independent of, routine obstetric care; women were explicitly informed that participation was voluntary and that declining would not affect in any way the care received. After providing written informed consent, participants completed the standardized digital protocol, either on site or at home. Recruitment was continuous over 15 months and spanned all calendar seasons, limiting—although not eliminating—potential seasonal effects on depressive symptomatology.

### 2.4 Participants

The sample comprised 774 pregnant women. Inclusion criteria: (a) age ≥18 years, (b) pregnancy from second trimester onward, (c) Italian language proficiency. Exclusion criteria: severe cognitive impairment or medical conditions preventing questionnaire completion. All participants provided written informed consent. A subsample of 200 women (25.8%) completed the postpartum follow-up (T1).

### 2.5 Follow-up procedure

At 6 months postpartum, women who had completed the initial antenatal assessment were re-contacted by telephone by project staff, who provided a direct link to the follow-up questionnaire. Two contact attempts were made, 2 weeks apart. Participation in this follow-up phase was voluntary and did not affect the continuity of clinical care. Where participants could not be reached, the reason for non-participation was systematically recorded, consisting primarily of failure to respond to contact attempts, outdated contact information, or explicit refusal to participate. Follow-up questionnaires were linked to the antenatal records by exact match on the participant code assigned at enrollment, after normalizing whitespace and punctuation within the code; no approximate or manual matching was performed. Of the 289 questionnaires returned, 76 carried a participant code that could not be matched to any record in the analyzed antenatal dataset and were therefore not used. The remaining questionnaires corresponded to 200 women; where two questionnaires were returned under the same participant code (*n* = 13), the first was retained, and all longitudinal results were unchanged when the second was used instead. The flow of participants through both assessment waves is summarized in Figure 1.

**Figure 1:**
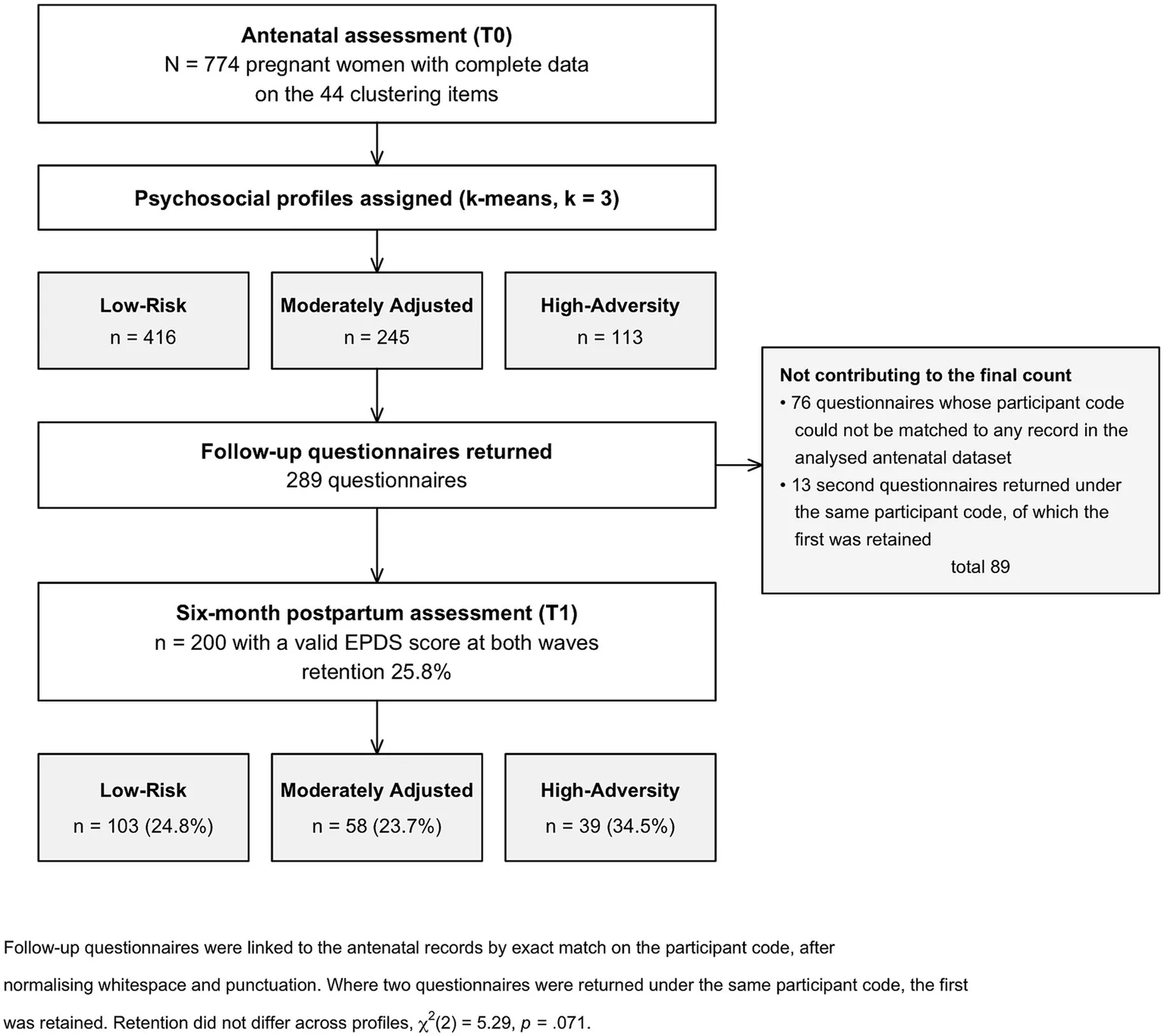
Participant flow diagram. Flow of participants from recruitment at the obstetric outpatient clinics through baseline assessment (T0, *N* = 774), profile assignment (Low-Risk *n* = 416; Moderately Adjusted *n* = 245; High-Adversity *n* = 113), and the 6-month postpartum assessment (T1, *n* = 200; retention 25.8%; Low-Risk 103, 24.8%; Moderately Adjusted 58, 23.7%; High-Adversity 39, 34.5%). Of the 289 questionnaires returned at follow-up, 76 carried a participant code that could not be matched to any record in the analyzed antenatal dataset; the remaining questionnaires corresponded to the 200 women shown, with two questionnaires returned under the same participant code in 13 cases, of which the first was retained.

### 2.6 Sample characteristics

The baseline sample comprised 774 pregnant women who completed the antenatal assessment (T0). The sociodemographic and clinical characteristics of the T0 sample (*N* = 774) are presented in Table 1. The mean age of participants was 32.65 years (SD = 5.43), with a range from 18 to 48 years. A substantial majority (89.7%) were married or cohabiting. Educational attainment was distributed as follows: 35.0% completed high school, 26.1% earned a bachelor’s degree, and 21.3% obtained postgraduate qualifications. Regarding employment, 50.1% were in stable employment, 22.1% were homemakers, and 15.4% were unemployed. Most assessments occurred during the third trimester (95.2%), with 62.9% at ≥37 weeks of gestation. Most pregnancies were planned (72.7%), conceived naturally (94.1%), and not classified as high-risk (73.6%).

**Table 1:** Sociodemographic and clinical characteristics of the sample at T0 (*N* = 774).

Characteristic	*N* (%) or *M* (SD)
Sociodemographic data	
Age (years), Mean (SD)	32.65 (5.43)
Relationship status	
Married/cohabiting	694 (89.7%)
Educational attainment	
High school diploma	271 (35.0%)
Bachelor’s degree	202 (26.1%)
Postgraduate qualification	165 (21.3%)
Employment status	
Stable employment	388 (50.1%)
Homemaker	171 (22.1%)
Unemployed	119 (15.4%)
Clinical & obstetric data	
Pregnancy trimester—third trimester	737 (95.2%)
Gestational week—≥37 weeks	487 (62.9%)
Pregnancy planning—planned	563 (72.7%)
Conception—natural	728 (94.1%)
Conception—medically assisted	46 (5.9%)
High-risk pregnancy—No	570 (73.6%)
High-risk pregnancy—Yes	204 (26.4%)
Parity—primiparous	471 (60.9%)
Antenatal classes—attended	349 (45.1%)

Two hundred and four women (26.4%) reported having a high-risk pregnancy. This variable was collected as a binary self-reported indicator (yes/no) within the study protocol; specific obstetric conditions underlying the perceived high-risk status were not further categorized. Forty-six pregnancies (5.9%) were conceived through medically assisted reproduction. Among participants, 211 (27.3%) reported that the pregnancy was unplanned.

Of the baseline sample, 200 participants completed the 6-month postpartum follow-up assessment (T1), representing a 25.8% retention rate. For this longitudinal subsample the mean age was 32.91 years (SD = 5.33); 94.5% were married or cohabiting, 67.0% were primiparous, 52.0% were in stable employment, and 23.0% had a high-risk pregnancy. The comparison with the women who did not complete the follow-up is reported in Supplementary Table S8.

### 2.7 Measures

Participants completed a comprehensive assessment including sociodemographic information and validated psychometric instruments.

The Edinburgh Postnatal Depression Scale (EPDS; Benvenuti et al., 1999; Cox et al., 1987) assessed depressive symptoms (10 items, *α* = 0.79). The Childhood Trauma Questionnaire–Short Form (CTQ-SF; Bernstein et al., 2003; Sacchi et al., 2018) evaluated early adversity across five domains (28 items, *α* > 0.87 for all subscales). Relational functioning was assessed using the Dyadic Adjustment Scale–7 (DAS-7; Hunsley et al., 2001; *α* > 0.83). The Maternity Social Support Scale (MSSS; Dabrassi et al., 2009; Webster et al., 2000) measured perceived support (6 items, *α* = 0.67). The Maternal Antenatal Attachment Scale (MAAS; Busonera et al., 2016; Condon, 1993) assessed prenatal bonding (19 items, *α* = 0.79).

### 2.8 Analytical strategy

To capture multidimensional psychosocial configurations, the analytical strategy prioritized a granular, item-level approach. Of the 60 candidate items—spanning the MSSS, CTQ-SF, MAAS, and DAS-7 scales—16 were excluded because their variance fell to zero after winsorization at the 5th/95th percentiles, since constant variables contribute nothing to the distance matrix while the retention of non-discriminating variables can mask cluster separation (Steinley and Brusco, 2008). These comprised the CTQ-SF Physical Abuse and Sexual Abuse subscales in their entirety and two Physical Neglect items, all showing extreme floor effects (95.5–99.2% of responses at the scale minimum), together with four MAAS items showing extreme ceiling effects (99.5–99.9% at the scale maximum). The remaining 44 items entered the cluster analysis. The full list and item-level variance statistics are reported in Supplementary Table S0. Items from the Edinburgh Postnatal Depression Scale (EPDS) were intentionally excluded from the clustering model to reserve this measure as an independent external criterion for clinical validation. Although KNN imputation (*k* = 5) had been planned for missing baseline data, this procedure proved unnecessary, as all 44 retained clustering items exhibited complete data. Outlier treatment (winsorization to 5th/95th percentiles), normality transformations (log₁₀ for variables with skewness > 1), and Z-score standardization were applied sequentially.

Because the CTQ-SF Minimization/Denial items (10, 16, and 22) constitute a validity index rather than a measure of maltreatment exposure, the clustering was repeated with these three items removed; the three-profile solution was recovered with high agreement (adjusted Rand index = 0.87; 95.1% concordant classification; Supplementary Table S2).

Reverse-coding of the positively worded CTQ-SF items and of the MAAS items requiring it under Condon’s original scoring had been applied at the dataset construction stage; the analytic pipeline did not further modify item directionality.

The optimal number of clusters was determined using convergent evidence from multiple criteria (Elbow, Silhouette, Gap statistic, and Bayesian Information Criterion via Gaussian Mixture Models), indicating a three-cluster solution. K-means clustering (*k* = 3) was performed with multiple random initializations. Cluster robustness was assessed through nonparametric bootstrap resampling (1,000 iterations), yielding high Jaccard similarity coefficients (0.81–0.93). Complete procedures are reported in the Supplementary materials.

To verify that the clustering solution was not driven by the unequal number of items across instruments, a sensitivity analysis was conducted on domain-level scores. Six domain scores (CTQ-SF emotional abuse, emotional neglect, and physical neglect; DAS-7, MSSS, and MAAS totals) were standardized and entered, with equal weight, into a k-means solution (*k* = 3). Agreement with the item-level solution was quantified using the adjusted Rand index and the proportion of concordantly classified participants. Full results are reported in Supplementary Table S1.

Because item-level scores were used to derive the clusters, between-profile differences on these items, and on the scale scores that aggregate them, are reported descriptively; no inferential tests are performed on the cluster-formation variables. Inferential analyses are restricted to external criteria that did not enter the clustering: the CTQ-SF Physical Abuse and Sexual Abuse subscales, EPDS scores, and sociodemographic and obstetric variables.

To evaluate the clinical relevance of the identified profiles, differences in depressive symptoms were examined cross-sectionally during pregnancy (T0) and longitudinally across the perinatal period using one-way ANOVA with cluster membership as the independent variable and EPDS total scores as the dependent measure, followed by Tukey HSD post-hoc comparisons; the baseline ANOVA was complemented by an adjusted linear model including age, education, employment, marital status, parity, pregnancy planning, mode of conception, and high-risk pregnancy status as covariates. The proportion of women scoring at or above the EPDS clinical cut-offs (≥ 9, the screening cut-off validated for the Italian population, Benvenuti et al., 1999; and ≥ 13, the more conservative international threshold) was compared across profiles with *χ*^2^ tests. Additionally, repeated-measures ANOVA was conducted with Time (T0, T1) as within-subject factor and Cluster membership as between-subject factor to assess whether the three psychosocial profiles were associated with differential trajectories of depressive symptomatology.

Attrition analysis. To evaluate whether follow-up non-response was selective, women who completed the T1 assessment were compared with non-completers on baseline EPDS scores, cluster membership, and sociodemographic and obstetric characteristics (independent-samples *t*-tests, *χ*^2^ tests, and a logistic regression predicting follow-up completion; Supplementary Table S8).

## 3 Results

### 3.1 Cluster composition and characterization

The clustering analysis identified three distinct psychosocial profiles during pregnancy, characterized by varying configurations of childhood trauma exposure, relational functioning, perceived social support, and maternal antenatal attachment. The three-cluster solution was validated by convergent clustering criteria and demonstrated high stability across bootstrap resampling procedures, with Jaccard similarity coefficients ranging from 0.81 to 0.93. A sensitivity analysis using equally weighted domain scores substantially reproduced the three-profile solution (adjusted Rand index = 0.76; 91.2% concordant classification; cluster sizes 417/254/103 vs. 416/245/113), indicating that the profiles reflect a stable multivariate structure rather than the larger number of CTQ-SF items (Supplementary Table S1). Mean subscale and scale scores across clusters are presented descriptively in Table 2, and the corresponding standardized profiles are shown in Figure 2.

**Table 2:** Psychosocial scale and subscale scores by profile (descriptive).

Variable, *M* (SD)	Low-Risk (*n* = 416)	Moderately Adjusted (*n* = 245)	High-Adversity (*n* = 113)
CTQ-SF^1^ total score (clinical scales)	26.33 (2.24)	28.44 (3.23)	43.58 (11.98)
Emotional abuse	5.29 (0.92)	5.61 (1.17)	10.37 (3.92)
Emotional neglect	5.63 (1.33)	7.00 (1.96)	13.46 (3.67)
Physical neglect	5.37 (1.09)	5.57 (1.19)	7.22 (2.38)
**Physical abuse**ᵃ	5.01 (0.20)	5.14 (0.54)	6.74 (3.94)
**Sexual abuse**ᵃ	5.03 (0.35)	5.12 (0.89)	5.79 (3.16)
**Minimization/Denial**ᵇ	12.53 (2.15)	11.49 (1.99)	8.58 (1.82)
MSSS^2^ total score	27.87 (2.01)	25.74 (2.42)	25.44 (2.84)
DAS-7^3^ total score	31.98 (2.73)	25.91 (4.69)	27.76 (5.31)
MAAS^4^ total score	84.82 (4.15)	79.00 (5.07)	80.83 (6.62)
Quality of attachment	48.26 (1.94)	45.74 (2.81)	46.27 (3.40)
Intensity of preoccupation	31.87 (3.17)	28.90 (3.34)	29.84 (4.01)
EPDS^5^ total score	4.05 (3.96)	7.43 (5.05)	8.32 (5.62)

^1^Childhood Trauma Questionnaire–Short Form; ^2^Maternity Social Support Scale; ^3^Dyadic Adjustment Scale–7; ^4^Maternal Antenatal Attachment Scale; ^5^Edinburgh Postnatal Depression Scale. All values were re-derived from the raw item-level data (*N* = 774; no missing values). Because these scores aggregate the items from which the profiles were derived, between-profile differences are reported descriptively, without inferential tests; the EPDS, which did not enter the clustering, is tested inferentially in the Results. ᵃThe Physical Abuse and Sexual Abuse subscales contributed no items to the cluster solution and function as external validators; because both show pronounced floor effects, they are compared through the proportion above the moderate-to-severe clinical cut-off rather than through their means (see Results and Supplementary Table S7). ᵇThe Minimization/Denial scale is a validity index rather than a measure of maltreatment exposure; it is reported for completeness and was excluded in the sensitivity analysis reported in Supplementary Table S2.

**Figure 2:**
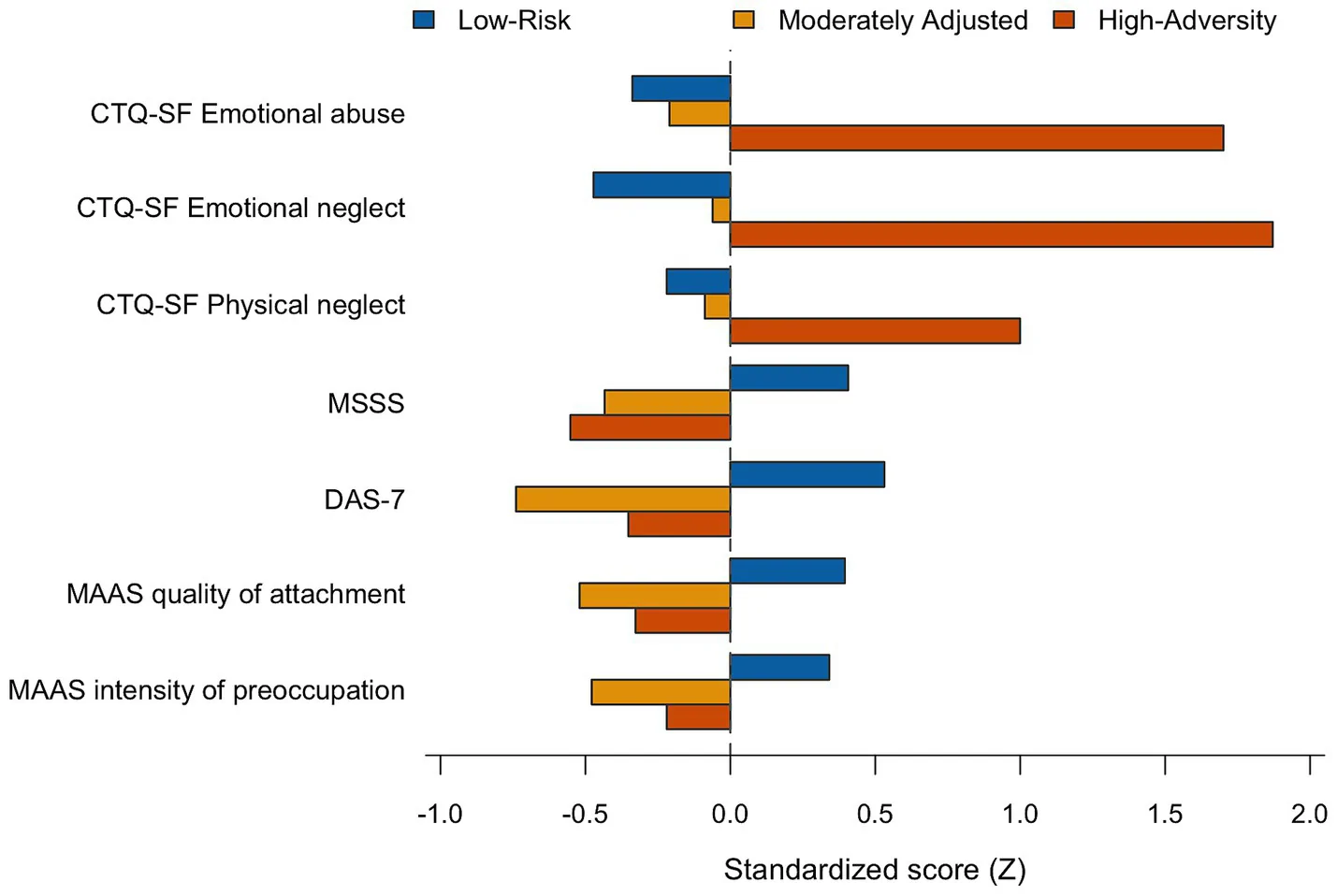
Standardized psychosocial profiles of the three clusters. Mean standardized (Z) scores on CTQ-SF Emotional Abuse, Emotional Neglect, and Physical Neglect (Physical and Sexual Abuse excluded owing to null variance after winsorization), MSSS, DAS-7, and the two MAAS subscales (quality of attachment and intensity of preoccupation), by profile. The dashed vertical line represents the sample mean (*Z* = 0). Values above zero indicate higher-than-average scores on the corresponding dimension; higher CTQ-SF scores indicate greater childhood adversity, whereas higher DAS-7, MSSS, and MAAS scores indicate greater current resources.

The Low-Risk profile (Cluster 3; 53.7%, *n* = 416) was marked by low levels of childhood trauma exposure, the highest perceived social support, the most favorable dyadic adjustment, and the highest antenatal attachment.

The Moderately Adjusted profile (Cluster 1; 31.7%, *n* = 245) exhibited intermediate levels of childhood adversity together with the least favorable current relational resources at the scale level: the lowest dyadic adjustment and the lowest total antenatal attachment across profiles, with perceived social support comparable to the High-Adversity profile.

The High-Adversity profile (Cluster 2; 14.6%, *n* = 113) was characterized by markedly the highest levels of childhood trauma exposure, together with low perceived social support (comparable to the Moderately Adjusted profile). At the scale level, dyadic adjustment and antenatal attachment totals were intermediate between the other two profiles; the item-level analyses reported below indicate that what distinguishes this profile is the trauma domain, whereas within antenatal attachment its reduction is concentrated in the items indexing the affective quality of the bond rather than being uniformly distributed.

### 3.2 Item-level characterization of psychosocial profiles

Because the clustering items themselves were used to derive the profiles, item-level patterns are reported descriptively (standardized means; see Supplementary Tables S3–S6), without inferential tests (see Analytical strategy).

Regarding childhood trauma, the High-Adversity profile stood well above both other profiles on every clinical CTQ-SF item, the largest between-profile deviations arising on the items indexing emotional neglect and emotional abuse. Items referring to the availability of a caring and protective family figure (negatively worded in the instrument and recoded so that higher scores denote greater adversity) separated the profiles most sharply: on these items (CTQ-SF 5, 7, 13, 19, and 28) the High-Adversity profile scored between 1.14 and 1.89 standard deviations above the sample mean, against values between −0.41 and −0.45 in the Low-Risk profile, whereas the Moderately Adjusted profile lay close to the sample mean throughout (*Z* range = −0.15 to +0.17). The physical neglect items showed the same ordering with smaller amplitude (High-Adversity *Z* = +0.30 to +1.40).

With respect to relational functioning, the Moderately Adjusted profile showed the lowest standardized score on every DAS-7 item, with the High-Adversity profile in an intermediate position and the Low-Risk profile uniformly above the sample mean (Supplementary Table S6). The two at-risk profiles converged on the item assessing overall relationship happiness (*Z* ≈ −0.54 in the Moderately Adjusted and ≈ −0.36 in the High-Adversity profile, against ≈ +0.42 in the Low-Risk profile), indicating that both share marked relational dissatisfaction despite their different trauma histories.

They diverged most on the items indexing agreement on aims, goals, and matters of importance to the couple, where the Moderately Adjusted profile fell to *Z* = −0.65 while the High-Adversity profile remained closer to the sample mean (*Z* = −0.24). Current couple functioning therefore does not track the severity of childhood adversity: at the item level as at the scale level, it is the Moderately Adjusted and not the High-Adversity profile that reports the least favorable dyadic adjustment.

Item-level analysis of antenatal attachment likewise revealed qualitatively distinct patterns across profiles. Descriptively, the High-Adversity profile showed reduced scores on items reflecting the affective quality of the bond—emotional closeness, positive emotional tone, and tenderness towards the fetus—while three items were the only ones on which this profile exceeded the Low-Risk profile: preoccupation with the baby, personification of the fetus as a real person, and the sense that the baby depends on the mother for its wellbeing (Supplementary Table S5). At the level of the instrument’s validated subscales this contrast is present but slight: relative to quality of attachment, intensity of preoccupation was higher by 0.11 SD in the High-Adversity profile and lower by 0.05 SD in the Low-Risk profile, a difference of 0.16 SD (Table 2; Figure 2). This pattern is explored further in the Discussion.

### 3.3 External validation: physical and sexual abuse

The CTQ-SF Physical Abuse and Sexual Abuse subscales contributed no items to the cluster solution—both were removed in their entirety at the item-selection stage—and therefore function as external validators. Because both subscales show pronounced floor effects, with a median at the scale minimum in every profile, mean comparisons are uninformative; the proportion of women at or above the moderate-to-severe clinical cut-off (Bernstein and Fink, 1998) was compared instead. For physical abuse, this proportion was 12.4% in the High-Adversity profile against 0.0% in both other profiles (14/113 vs. 0/245 and 0/416; *χ*^2^(2) = 83.40, *p* < 0.001); for sexual abuse, 8.0% against 1.6 and 0.5% (9/113 vs. 4/245 and 2/416; *χ*^2^(2) = 26.36, *p* < 0.001; Supplementary Table S7). The clustering thus identified women with greater physical and sexual abuse exposure without those items having entered the analysis.

### 3.4 Depressive symptoms during pregnancy (T0)

Between-profile differences in depressive symptoms at T0 were significant (*F*(2, 771) = 62.52, *p* < 0.001, *η*^2^ = 0.140), with both the Moderately Adjusted and the High-Adversity profiles scoring higher than the Low-Risk profile (Tukey HSD, both *p* < 0.001; raw EPDS means 4.05, 7.43, and 8.32 for the Low-Risk, Moderately Adjusted, and High-Adversity profiles, respectively).

The association between cluster membership and depressive symptoms remained essentially unchanged after adjustment for sociodemographic and obstetric covariates (*F*(2, 747) = 58.15, *p* < 0.001, partial *η*^2^ = 0.135). Adjusted marginal means (4.06, 7.40, and 8.33) closely matched the unadjusted values, and both at-risk profiles differed significantly from the Low-Risk profile (*b* = +3.34 and *b* = +4.27, respectively; both *p* < 0.001). Only marital status was independently associated with symptoms (*p* = 0.004). Full results are reported in Supplementary Table S9. Exploratory comparisons of EPDS scores and profile distribution across obstetric subgroups are reported in Supplementary Table S12. Depressive symptoms were slightly higher in women with an unplanned pregnancy (*M* = 6.33 vs. 5.52; *p* = 0.055, *d* = −0.16) and in multiparous women (*M* = 6.23 vs. 5.42; *p* = 0.034, *d* = −0.16), and did not differ by high-risk status (*p* = 0.384) or mode of conception (*p* = 0.084). Profile distribution differed only by pregnancy planning, with the High-Adversity profile more frequent among unplanned pregnancies (19.4% vs. 12.8%; *χ*^2^(2) = 6.25, *p* = 0.044, *V* = 0.09); it did not differ by high-risk status (*p* = 0.972), parity (*p* = 0.165), or mode of conception (*p* = 0.224). These comparisons are unadjusted and exploratory; the adjusted model above addresses the same substantive question.

The proportion scoring above the EPDS clinical cut-off (≥ 9) increased across profiles: at T0, 11.1% of Low-Risk, 35.9% of Moderately Adjusted, and 44.2% of High-Adversity women exceeded the threshold (*χ*^2^(2) = 83.20, *p* < 0.001), with a comparable gradient using ≥ 13 (5.0, 15.5, 24.8%; *χ*^2^(2) = 41.22, *p* < 0.001) (Table 3).

**Table 3:** Depressive symptoms by profile across the perinatal period.

EPDS score	Panel A: EPDS among completers of both waves (*n* = 200)		
	Low-Risk (*n* = 103)	Moderately Adjusted (*n* = 58)	High-Adversity (*n* = 39)
EPDS T0, M (*SD*)	3.79 (3.50)	6.81 (4.11)	8.97 (5.98)
EPDS T1, M (*SD*)	4.56 (4.66)	6.64 (4.97)	8.31 (4.69)

EPDS cut-off	Panel B: Proportion above clinical EPDS cut-offs at T0 (*N* = 774)				
	Low-Risk (*n* = 416)	Moderately Adjusted (*n* = 245)	High-Adversity (*n* = 113)	*χ*^2^(2)	*p*
EPDS ≥ 9, % (*n*)	11.1 (46)	35.9 (88)	44.2 (50)	83.20	< 0.001
EPDS ≥ 13, % (*n*)	5.0 (21)	15.5 (38)	24.8 (28)	41.22	< 0.001

EPDS cut-off	Panel C: Proportion above clinical EPDS cut-offs at T1 (*n* = 200)				
	Low-Risk (*n* = 103)	Moderately Adjusted (*n* = 58)	High-Adversity (*n* = 39)	*χ*^2^(2)	*p*
EPDS ≥ 9, % (*n*)	18.4 (19)	32.8 (19)	46.2 (18)	11.69	0.003
EPDS ≥ 13, % (*n*)	7.8 (8)	10.3 (6)	15.4 (6)	1.83	0.40

EPDS, Edinburgh Postnatal Depression Scale. Cut-offs: ≥ 9 (screening cut-off validated for the Italian population; Benvenuti et al., 1999) and ≥ 13 (probable depression, international threshold). Panels A and C refer to the 200 women with a valid EPDS score at both waves, so that the rows are directly comparable; the repeated-measures analysis on these data is reported in Supplementary Table S11. Panel B refers to the full baseline sample. Follow-up records were linked to the antenatal dataset by exact match on the participant code, after normalizing whitespace and punctuation; the procedure is described in the Methods and its outcome is summarized in Figure 1.

### 3.5 Attrition analysis

Follow-up completion did not differ significantly across profiles: 24.8% (103/416) in the Low-Risk, 23.7% (58/245) in the Moderately Adjusted, and 34.5% (39/113) in the High-Adversity profile (*χ*^2^(2) = 5.29, *p* = 0.071, Cramér’s *V* = 0.08)—if anything, retention was highest in the most burdened profile, the opposite of what severity-driven attrition would produce. Completers and non-completers did not differ in baseline depressive symptoms, overall (*M* = 5.67, SD = 4.72 vs. *M* = 5.76, SD = 5.03; *p* = 0.825, *d* = −0.02) or within any profile (all *p* ≥ 0.29), nor in high-risk pregnancy status (*p* = 0.211), pregnancy planning (*p* = 0.409), mode of conception (*p* = 0.699), or gestational week (*p* = 0.097). Attrition was instead patterned by engagement with services and by social resources: completers were more likely to have attended antenatal classes (58.0% vs. 40.6%; *χ*^2^(1) = 18.15, *p* < 0.001), to hold a university degree (56.0% vs. 44.4%; *χ*^2^(1) = 7.97, *p* = 0.005), to be married or cohabiting (94.5% vs. 88.0%; *χ*^2^(1) = 6.81, *p* = 0.009), and to be primiparous (67.0% vs. 58.7%; *χ*^2^(1) = 4.28, *p* = 0.039). In a logistic regression, neither baseline EPDS (OR = 0.99, *p* = 0.686) nor cluster membership predicted completion, whereas antenatal-class attendance (OR = 1.85, *p* = 0.002) and marital status (OR = 2.00, *p* = 0.045) did (Supplementary Table S8).

### 3.6 Depressive symptoms across the perinatal period by cluster membership

To investigate the longitudinal effects of cluster membership, a mixed repeated-measures ANOVA was performed on the subsample of participants with complete EPDS data at both assessment points (*n* = 200). The analysis identified a significant main effect of Cluster (*F*(2, 197) = 22.74, *p* < 0.001, partial *η*^2^ = 0.188), indicating consistent between-group differences in depressive symptom severity throughout the perinatal period. Neither the main effect of Time (*F*(1, 197) = 0.38, *p* = 0.54) nor the Cluster × Time interaction (*F*(2, 197) = 1.40, *p* = 0.25) achieved statistical significance, indicating generally parallel symptom trajectories from pregnancy (T0) to 6 months postpartum (T1). Within this subsample, mean EPDS scores were 3.79 (SD = 3.50) at T0 and 4.56 (SD = 4.66) at T1 in the Low-Risk profile, 6.81 (SD = 4.11) and 6.64 (SD = 4.97) in the Moderately Adjusted profile, and 8.97 (SD = 5.98) and 8.31 (SD = 4.69) in the High-Adversity profile (Table 3; Figure 3; Supplementary Table S11). Post-hoc comparisons elucidated the nature of these between-group differences. During pregnancy the three profiles were ordered and mutually distinct, each at-risk profile scoring above the Low-Risk one (Moderately Adjusted *p* < 0.001; High-Adversity *p* < 0.001) and the High-Adversity profile also above the Moderately Adjusted one (*p* = 0.040). At 6 months postpartum both at-risk profiles remained significantly elevated relative to the Low-Risk profile (High-Adversity *p* < 0.001; Moderately Adjusted *p* = 0.023), while the two no longer differed from each other (*p* = 0.210). The clinical gradient was mirrored by the proportions above the screening cut-off at T1 (EPDS ≥ 9: 18.4, 32.8, and 46.2% in the Low-Risk, Moderately Adjusted, and High-Adversity profiles; *χ*^2^(2) = 11.69, *p* = 0.003); at the ≥ 13 threshold the ordering was preserved (7.8, 10.3, 15.4%) but the comparison was not significant (*χ*^2^(2) = 1.83, *p* = 0.40), reflecting the small cell counts at the more conservative threshold. The absence of significant Time and Cluster × Time effects does not indicate remission of risk, but rather the persistence of stable between-group differences across the perinatal period. Cluster membership identified during pregnancy therefore remained associated with depressive symptom severity over time, indicating that antenatal psychosocial vulnerability may persist beyond childbirth rather than resolving spontaneously (Table 3; see also Supplementary Tables S10, S11).

**Figure 3:**
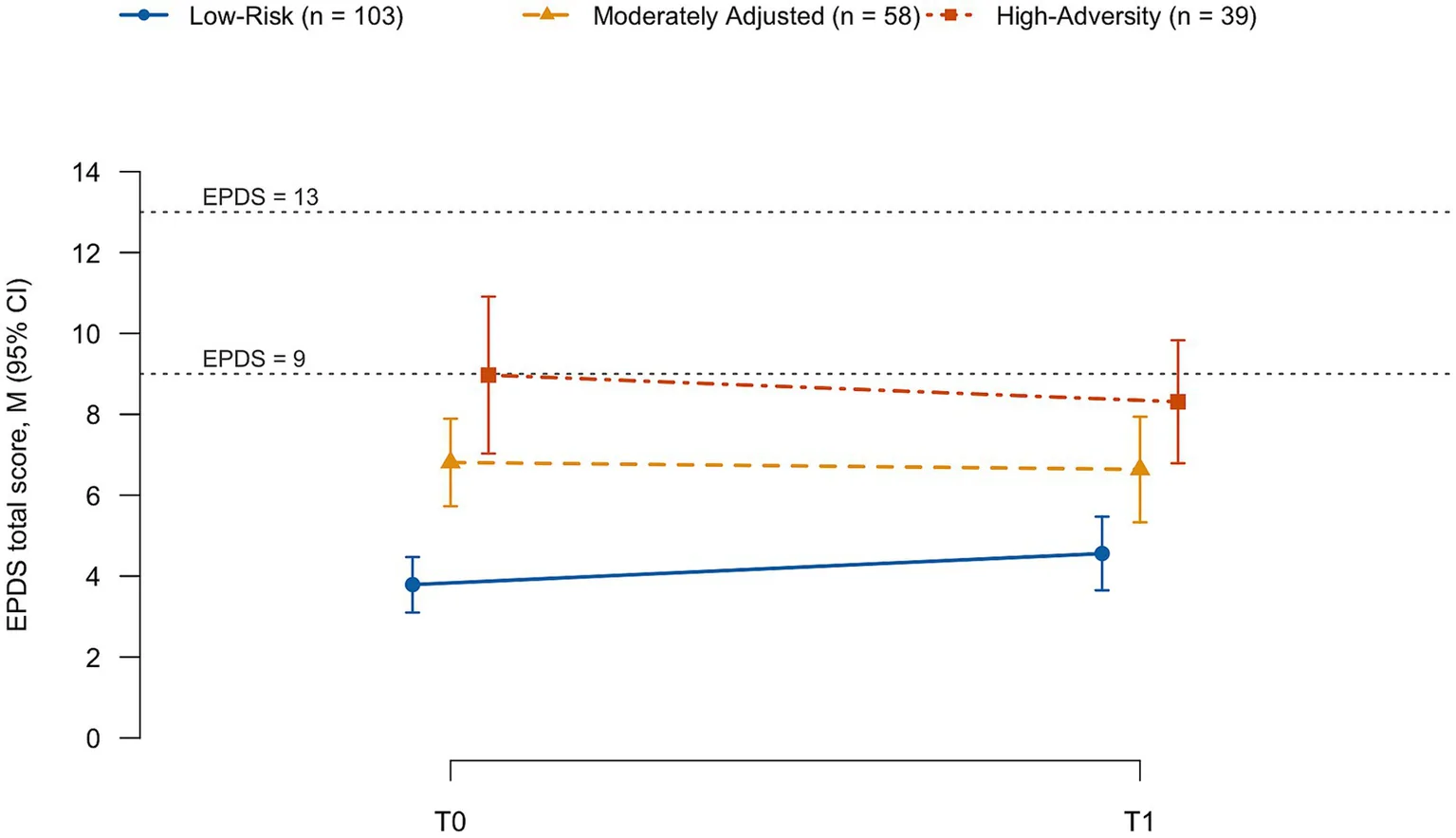
Depressive symptoms across the perinatal period by profile. Mean EPDS scores (with 95% confidence intervals) at T0 (pregnancy) and T1 (6 months postpartum) among the 200 completers (Low-Risk 103, Moderately Adjusted 58, High-Adversity 39), by profile. Dotted horizontal lines mark the clinical cut-offs (EPDS ≥ 9 and ≥ 13). Because the EPDS distribution is right-skewed, profile means remain below the screening threshold even where a substantial minority of women exceeds it; the corresponding proportions are reported in Table 3, Panel C.

## 4 Discussion

This study identified three distinct psychosocial-clinical profiles during the perinatal period: Low-Risk, Moderately Adjusted, and High-Adversity. Notably, the analytical approach prioritized item-level examination over total scale scores, enabling the detection of clinically meaningful heterogeneity that aggregate measures often obscure. Cluster membership determined during pregnancy effectively stratified risk for postpartum depressive symptoms, and between-profile differences in symptom severity persisted across the perinatal period.

### 4.1 The protective triad: support, partnership, and bonding

The Low-Risk profile consistently exhibited low depressive symptoms, remaining below clinical thresholds at both timepoints. This group reported optimal conditions across all dimensions: highest perceived social support and dyadic adjustment, strongest positive prenatal attachment, and lowest childhood trauma exposure. These findings underscore the synergistic role of a “protective triad”—high social support, strong partnership quality, and early positive bonding—in buffering women against postnatal depressive symptoms (Wierenga et al., 2025; Wohrer et al., 2024). In this sense, the Low-Risk profile can be read as the person-centered signature of relational resilience: the joint configuration of low developmental adversity and supportive current relationships mirrors the buffering and mediating role that resilience—particularly in its relational forms—plays in the pathway from childhood adversity to perinatal depression (Young-Wolff et al., 2019; Howell et al., 2020). The convergence of minimal developmental adversity with robust current psychosocial resources creates a Low-Risk profile characterized by sustained emotional wellbeing. Conversely, the High-Adversity group maintained the highest depressive symptom levels at both timepoints, differing significantly from the Low-Risk group throughout. This pattern of persistent vulnerability is underpinned by the highest levels of childhood emotional and physical neglect/abuse, paired with low perceived social support—comparable, at the scale level, to the Moderately Adjusted profile—while current couple functioning, although less favorable than in the Low-Risk profile, was not the most impaired of the three. This pattern suggests that a history of developmental trauma, coupled with a lack of protective adult relational resources, significantly predisposes women to persistent perinatal depressive symptomatology (Choi et al., 2022; Tebeka et al., 2021).

### 4.2 Differential vulnerability: qualitative distinction of at-risk profiles

Both at-risk profiles remained elevated relative to the Low-Risk profile across the perinatal period, and neither showed a systematic change in symptom level between pregnancy and 6 months postpartum. What changed was the distance between them: during pregnancy the three profiles were mutually distinct, whereas at follow-up the Moderately Adjusted and High-Adversity profiles no longer differed from each other, both remaining above the Low-Risk profile. Although the Cluster × Time interaction did not achieve statistical significance, item-level analysis revealed that these parallel trajectories obscure qualitatively distinct configurations of developmental adversity and relational functioning—differences that aggregate scores alone could not capture.

The High-Adversity group showed marked deficits on items reflecting the availability of protective caregivers during childhood. CTQ items that address the presence of someone who provided care, protection, and support (e.g., CTQ-SF items 2, 7, and 13) showed standardized scores between 1.4 and 1.8 standard deviations above the sample mean on the reverse-coded dimension, in stark contrast to Low-Risk women who scored well below the mean. Item 26 (“There was someone to take me to the doctor if I needed it”) separated the High-Adversity group in the same direction, though less sharply (*Z* = +0.91 against −0.29 in the Low-Risk profile), indicating physical neglect: not just material deprivation, but a fundamental absence of a protective figure who could be relied upon for safety and care (Choi et al., 2017). These data suggest that the absence of protective and reliable caregivers during childhood represents a fundamental disruption in developmental security, which has repercussions throughout life and profoundly affects the capacity for trust, emotional regulation, and the ability to build secure adult relationships (Choi et al., 2017). This form of neglect is associated with more pervasive and treatment-resistant psychological difficulties than other forms of adversity, as it strikes at the core of attachment security and the development of internal working models (Infurna et al., 2024).

The differentiation extends beyond the severity of trauma to the way current relational resources are configured. The Moderately Adjusted group was characterized by a broad impairment of couple functioning: it reported the lowest standardized score on every DAS-7 item and the lowest DAS-7 total, with dissatisfaction on the global happiness item comparable to that of the High-Adversity group. This suggests that prenatal distress in this group may be sustained largely by a current emotional stressor, diminished perceived relationship quality, in the relative absence of severe developmental adversity (Rajendran et al., 2024). The impairment was most marked on DAS item 2, which assesses core relationship structure—agreement on aspirations, goals, and important life matters—pointing to a misalignment in shared life vision (Çankaya and Alan Dikmen, 2022) rather than to dissatisfaction alone. The High-Adversity group, by contrast, combined by far the highest burden of childhood maltreatment with an intermediate position on current dyadic indicators: developmental adversity and current couple functioning did not coincide, and it is precisely this dissociation that a person-centered, item-level reading makes visible.

The same ordering held for perceived partner support: the Moderately Adjusted group reported the least instrumental help from the partner (MSSS item 3, *Z* = −0.36, against −0.10 in the High-Adversity and +0.24 in the Low-Risk profile), so that practical assistance and structural couple alignment (DAS items 2 and 7) moved together rather than dissociating. This profile is thus defined by a convergent weakening of the current relational field—less agreement on shared goals, less practical support, lower global satisfaction—while its developmental history remains close to that of the Low-Risk profile. This reading aligns with the literature on secure base functioning in romantic relationships. Feeney and Collins (2015) argue that the defining feature of a functional adult partnership is not transient satisfaction, but the partners’ capacity to support each other’s exploration, goal pursuit, and future-oriented personal growth. Relational quality is grounded in the relationship’s ability to sustain meaning and development—not merely in affective comfort. A shared future vision, therefore, indexes a structural dimension of the couple’s system: alignment of values, intentions, and planned trajectories enabling both partners to maintain identity continuity and psychological agency over time—the dimension on which the Moderately Adjusted profile is most clearly wanting.

### 4.3 The configuration of antenatal attachment under adversity

At the scale-total level, antenatal attachment did not sharply distinguish the two at-risk profiles: the High-Adversity total was slightly above the Moderately Adjusted one, and both were below the Low-Risk profile (Table 2). The item-level analysis, however, revealed a distinctive internal configuration within the High-Adversity profile: reduced scores on the items indexing the affective quality of the bond, alongside comparatively preserved scores on the items indexing preoccupation with, and representational personification of, the fetus—the only three items on which this profile exceeded the Low-Risk profile—a configuration in which affective bonding appears attenuated while certain representational components remain relatively active (Howard and Khalifeh, 2020). One possible clinical reading of this pattern—to be tested directly in future work—draws on the notion of a predominantly cognitive engagement with the fetus in the context of trauma described by Imbasciati and Cena (2023): some mothers, particularly those with a trauma history, may form distinctly cognitive and rationalized relationships with their unborn children, involving intense mental preoccupation and the attribution of thoughts, intentions, and identity to the baby, in the relative absence of affective resonance; on this reading, cognitive investment would serve a defensive function, shielding mothers from the perceived risks of emotional closeness. This framework converges with findings from Choi et al. (2017), which demonstrate that maternal childhood trauma predicted postpartum depressive symptoms not merely through increased negative affect, but via specific avoidant affective processing mechanisms: trauma-exposed mothers were less able or willing to engage with emotionally salient stimuli, and this “affective dampening” partially mediated the trauma–postpartum psychopathology link. We advance this as a hypothesis rather than an established mechanism, and its evidential basis should be stated plainly. The observation rests on a small number of items rather than on validated subscales; the MAAS items indexing affective quality are negatively worded and reverse-scored, so that their distribution is more compressed at the scale ceiling than that of the remaining items; and at the level of Condon’s validated subscales the dissociation, although present in the expected direction, is slight (0.16 SD between the High-Adversity and Low-Risk profiles), with both quality of attachment and intensity of preoccupation reduced in the two at-risk profiles. The present data are therefore consistent with this reading but do not establish it, and dedicated testing—with measures of prenatal mentalization designed for the purpose—is required before it can be sustained.

### 4.4 Findings in the context of previous literature

Our findings converge with, and extend, previous person-centered evidence. The proportion of women in the High-Adversity profile (14.6%) and the graded pattern of depressive risk across profiles are consistent with recent latent-profile evidence in pregnant women exposed to childhood maltreatment, in which distinct resilient and vulnerable configurations of psychological, maternal, and interpersonal functioning have been identified (Legendre et al., 2026), and with person-centered analyses combining early adversity and current relational risk, in which profiles defined by cumulative adversity showed the poorest psychosocial functioning during pregnancy (Goldstein et al., 2020). At the level of specific, non-aggregated findings, the centrality of emotional and physical neglect items in differentiating the High-Adversity profile echoes longitudinal evidence identifying neglect as a specific predictor of perinatal depressive symptoms (Li et al., 2017; Tebeka et al., 2021), while the discriminant role of relationship satisfaction and dyadic alignment is in line with evidence on couple functioning and perinatal depression in Italian and international samples (Çankaya and Alan Dikmen, 2022; Molgora et al., 2019). Within the Italian context, the levels and distribution of antenatal depressive symptoms observed here are comparable to those reported by national and multicenter studies (Banti et al., 2011; Giardinelli et al., 2012; Cena et al., 2021), supporting the representativeness of our clinical sample while highlighting the added value of profiling over prevalence estimation.

### 4.5 Limitations

Several limitations should be acknowledged, together with their implications for the interpretation of the present findings and the strategies adopted—or recommended—to mitigate them. First, only a quarter of the baseline sample (25.8%) completed the postpartum assessment. Beyond reducing statistical power for longitudinal contrasts, selective attrition could bias estimates of symptom persistence: had women with greater psychosocial burden been less likely to respond, postpartum symptom levels—particularly in the High-Adversity profile—would be underestimated. The attrition analyses attenuate this concern: retention did not differ significantly across profiles and was, if anything, highest in the High-Adversity profile (34.5%); completers did not differ from non-completers in baseline symptomatology, overall or within any profile; and neither baseline symptoms nor profile membership predicted completion. Attrition was instead patterned by engagement with services and by social resources—antenatal-class attendance, education, marital status, and parity—indicating that the longitudinal subsample over-represents more educated, more engaged, more supported women. Nonetheless, the longitudinal findings should be considered preliminary, and future waves of the project will adopt scheduled multi-channel reminders and flexible administration to improve retention.

Second, the item-level characterization of the profiles is descriptive and exploratory. Because the same items were used to derive the clusters, inferential testing on those items would be circular; between-profile inference was therefore restricted to external criteria (the EPDS and the excluded Physical and Sexual Abuse subscales). Item-level patterns should accordingly be treated as hypothesis-generating until replicated in independent samples, ideally with preregistered analyses. The item-selection criterion carries a further limitation. Because the Physical Abuse and Sexual Abuse items showed no variance after winsorization (a consequence of their low prevalence in a community antenatal sample) both subscales were excluded from the clustering, so that two forms of maltreatment with high clinical impact did not contribute to profile derivation. In principle this could have concealed a subgroup defined primarily by severe physical or sexual abuse. The external validation argues against that possibility in the present sample: women scoring at or above the moderate-to-severe cut-off for physical abuse (12.4%) and sexual abuse (8.0%) were concentrated in the High-Adversity profile, against 0.0 and 1.6% in the Moderately Adjusted and 0.0 and 0.5% in the Low-Risk profile (both *p* < 0.001; Supplementary Table S7). The methodological limitation nonetheless stands, and samples enriched for severe maltreatment may yield a profile structure that this criterion would not recover. Third, all constructs were assessed via self-report, introducing shared method variance, and childhood experiences were assessed retrospectively. Retrospective reports of maltreatment show only partial agreement with prospective records (Baldwin et al., 2019); at the same time, subjective appraisals of early adversity are robustly and independently associated with psychopathology (Danese and Widom, 2020) and are arguably the clinically relevant index for prevention-oriented screening. Multi-informant and interview-based assessment would nonetheless strengthen future studies.

Fourth, information on the circumstances of unplanned pregnancies and on the characteristics of medically assisted conceptions was limited, constraining the depth with which these potentially higher-risk subgroups could be characterized; high-risk pregnancy was likewise recorded as a single binary indicator, so that the specific obstetric conditions underlying it could not be examined separately, and the exploratory subgroup comparisons we report should be read with this coarseness in mind. Fifth, follow-up was limited to 6 months postpartum, precluding conclusions about longer-term maternal trajectories and infant developmental outcomes. The design also constrains what the longitudinal analysis can establish: between-profile differences in depressive symptoms persisted across the perinatal period, but profile membership itself was not shown to be stable. Profiles were derived from antenatal data only, and women were not re-classified at follow-up; whether individuals moved between configurations after childbirth is an open question that would require repeated assessment of all four domains and a latent transition framework. Finally, recruitment took place at a single, large obstetric center in Southern Italy: cultural factors—including family structure and informal support networks—may shape both the composition and the prevalence of psychosocial profiles, limiting generalizability to other cultural and healthcare contexts, although the single-site design ensured procedural homogeneity across participants. Replication across regions, countries, and care systems is warranted.

Despite these limitations, the study provides robust cross-sectional evidence for the existence of distinct psychosocial profiles during pregnancy, while highlighting important directions for future longitudinal and multimethod research.

### 4.6 Clinical implications

Pregnancy constitutes a pivotal period of plasticity and therapeutic potential. The present findings highlight that perinatal mental health trajectories are influenced not solely by symptom severity but are embedded within qualitatively distinct developmental profiles, relational configurations, and representational organizations of the fetus, which can only be comprehensively understood through detailed, item-level assessment. The severity of childhood trauma is pivotal in differentiating between Low-Risk and chronic risk trajectories, impacting both couple functioning dynamics and antenatal attachment configuration. Risk assessment during pregnancy should encompass not only symptom screening but also the severity and patterning of childhood trauma and micro-level relational indicators, such as dyadic alignment, shared future meaning, and multidimensional antenatal attachment. The item-level approach demonstrates that aggregate scores alone are insufficient for capturing specific vulnerabilities that determine clinical trajectories. There is a need for stratified clinical pathways: transient affective dysregulation linked to relational dissatisfaction, as observed in Moderately Adjusted cases, may respond effectively to short-term supportive interventions aimed at enhancing couple communication and relationship satisfaction. Conversely, the High-Adversity configuration—severe developmental adversity carried into pregnancy, with current relational resources that are intermediate rather than protective—likely requires trauma-informed interventions emphasizing the integration of affect, representation, and relational security. Identifying configurations of cognitive personification in the relative absence of affective connection during gestation—should this pattern be confirmed by dedicated studies—may facilitate targeted, trauma-informed interventions at a time when trajectories are not yet crystallized. Multidimensional assessment of antenatal attachment is a clinical priority for timely and preventive perinatal mental health care. The item-level approach is not an analytic refinement but a clinical necessity: aggregate scores obscure the very heterogeneity (structural or affective relational deficits, protective or absent caregiving histories, distinct configurations of prenatal attachment) that determines whether vulnerability resolves spontaneously or crystallizes into chronic impairment.

## Data Availability

The datasets presented in this article are not readily available because they contain sensitive health information on pregnant and postpartum women. Requests to access the datasets should be directed to Maria Rita Infurna, mariarita.infurna@unipa.it.
