# Supplementary material for: Past wounds, present bonds, future mothers: item-level analysis of perinatal risk profiles
Source: Front Psychol. 2026 Oct 9;17:1899047. doi: 10.3389/fpsyg.2026.1899047 (PMC13690512; doi:10.3389/fpsyg.2026.1899047)
Supplement: Supplementary file 1 [file Presentation_1.pdf]

Supplementary Material

Past Wounds, Present Bonds, Future Mothers: Item-Level Analysis of Perinatal Risk Profiles

What this supplement contains

This supplement contains: (1) extended analytical procedures for the item-level clustering, including the item-selection criterion and the two sensitivity analyses conducted during revision; (2) the complete item-wise characterisation of the three profiles across all four instruments: childhood trauma (CTQ-SF), relational functioning (DAS-7), social support (MSSS), and antenatal attachment (MAAS), reported descriptively; (3) the external validation of the profiles on measures that did not contribute to their derivation; (4) the attrition analysis comparing completers and non-completers of the postpartum assessment; and (5) the record linkage between the two assessment waves, together with the cross-sectional and longitudinal associations between profile membership and EPDS scores, unadjusted and adjusted for sociodemographic and obstetric covariates, together with exploratory comparisons across obstetric subgroups. Throughout, profiles are presented in the order Low-Risk, Moderately Adjusted, High-Adversity, as in the main text.

1 Supplementary Methods

1.1 M1. Extended analytical procedure

The clustering strategy adopted an item-level, person-centred approach to capture multidimensional psychosocial configurations during pregnancy. The initial pool comprised 60 items from the MSSS, CTQ-SF, MAAS, and DAS-7 instruments. Items entered the analysis individually; aggregate and subscale scores were not used as clustering variables. EPDS items were intentionally excluded from the clustering solution to preserve depressive symptoms as an independent external clinical validator.

Sixteen of the 60 candidate items were excluded on a single, reproducible criterion: their variance fell to zero after winsorisation at the 5th/95th percentiles, indicating that they carried no information for a distance-based solution. These comprised the CTQ-SF Physical Abuse (items 9, 11, 12, 15, 17) and Sexual Abuse (items 20, 21, 23, 24, 27) subscales in their entirety, two Physical Neglect items (4 and 6), and four MAAS items (12, 15, 16, 19). The pattern differs by instrument: the excluded CTQ-SF items show extreme floor effects, with 95.5–99.2% of participants at the scale minimum, as expected for physical and sexual abuse in a community antenatal sample, whereas the excluded MAAS items show extreme ceiling effects, with 99.5–99.9% at the scale maximum. The remaining 44 items entered the cluster analysis. Descriptive statistics, raw and post-winsorisation variances, and the percentage of extreme responses for all 60 candidate items are reported in Table S0, making the selection fully reproducible from the dataset.

Although KNN imputation (k = 5) had been planned for missing baseline data, this procedure proved unnecessary: all 44 retained clustering items showed complete data (N = 774, no missing values). The preprocessing pipeline then applied, in sequence: (1) winsorisation of extreme values to the 5th/95th percentiles; (2) $\log_{10}$ transformation for variables with skewness > 1; and (3) Z-score standardisation to place all items on a common scale prior to distance-based clustering. Reverse-coding of the positively worded CTQ-SF items and of the MAAS items requiring it per Condon's original scoring had been applied at the dataset construction stage; the analytic pipeline did not further modify item directionality.

The optimal number of clusters was determined using convergent evidence from four criteria applied simultaneously: the Elbow method, the Silhouette method, the Gap statistic, and the Bayesian Information Criterion derived from Gaussian Mixture Models. All four criteria indicated a three-cluster solution. K-means clustering (k = 3) was subsequently performed with multiple random initialisations to minimise sensitivity to starting values. Cluster robustness was assessed via nonparametric bootstrap resampling (1,000 iterations); Jaccard similarity coefficients ranged from 0.81 to 0.93, above the conventional ≥ .75 threshold for cluster stability (Hennig, 2007), indicating high stability of the final solution across all three clusters.

1.2 M2. Sensitivity analyses

Two sensitivity analyses were conducted during revision.

First, to verify that the solution was not driven by the unequal number of items contributed by each instrument (the CTQ-SF contributes 16 of the 44 retained items, the MAAS 15, the MSSS 6, and the DAS-7 seven) the clustering was repeated on domain-level scores with equal weighting. Six standardised domain scores (CTQ-SF emotional abuse, emotional neglect, and physical neglect, computed on the retained items; DAS-7, MSSS, and MAAS totals) each entered a k-means solution (k = 3) with weight 1/6. Agreement with the primary solution was quantified through the adjusted Rand index and the proportion of concordantly classified participants (Table S1). Because the CTQ-SF still contributes three of the six domains under this scheme, a second, strictly instrument-balanced solution was also computed, in which each instrument contributed a single standardised score (CTQ-SF, DAS-7, MSSS, MAAS) carrying one quarter of the weight.

Second, because the CTQ-SF Minimization/Denial items (10, 16, and 22) constitute a validity index rather than a measure of maltreatment exposure, the clustering was repeated with these three items removed. Two complementary analyses are reported: re-clustering at item level on the remaining 41 items, and the domain-level solution described above run with and without a Minimization/Denial domain (Table S2).

1.3 M3. Descriptive characterisation and external validation

Because the item-level scores were used to derive the clusters, between-profile differences on those items and on the scale scores that aggregate them are reported descriptively, without inferential testing, which under these conditions would be circular. Tables S3–S6 therefore report standardised means (Z-scores computed on the winsorised item pool, $M = 0$, $SD = 1$ across the full sample) together with a descriptive index of separation, defined as the range of the three profile means expressed in standard-deviation units. This replaces the Kruskal–Wallis and Dunn tests reported in the previous version of this supplement.
Inferential analyses are restricted to variables that did not contribute to profile derivation: the CTQ-SF Physical Abuse and Sexual Abuse subscales, EPDS scores, and sociodemographic and obstetric characteristics. The two excluded CTQ-SF subscales constitute a non-circular validation of the solution, since neither contributed any item to the clustering. Because both show pronounced floor effects, with a median at the scale minimum in every profile, their means are not compared: under a floor of this kind the between-group F is algebraically equivalent to Levene's test of variance homogeneity and would not speak to a difference in location. The proportion of women at or above the moderate-to-severe clinical cut-off (Bernstein & Fink, 1998) is reported instead, as it poses the clinically meaningful question and is robust to the floor (Table S7).

1.4 M4. Attrition analysis

At 6 months postpartum, women who had completed the antenatal assessment were re-contacted by telephone by project staff, who provided a direct link to the follow-up questionnaire; two contact attempts were made, two weeks apart, without financial incentives. Where participants could not be reached, the reason for non-participation was systematically recorded, consisting primarily of failure to respond to contact attempts, outdated contact information, or explicit refusal to participate. Returned questionnaires were linked to the antenatal dataset by exact match on the participant code assigned at enrolment, after normalising whitespace and punctuation within the code; no approximate or manual matching was performed. Of the 289 questionnaires returned, 76 carried a participant code that could not be matched to any record in the analysed antenatal dataset and were therefore not used. The remaining questionnaires corresponded to 200 women; where two questionnaires were returned under the same participant code ($n = 13$), the first was retained, and all longitudinal results were unchanged when the second was used instead. The procedure yielded 200 women (25.8% of the baseline sample) with a valid EPDS score at both waves.
To evaluate whether follow-up non-response was selective, completers were compared with non-completers on baseline EPDS scores (overall and within each profile), profile membership, and all sociodemographic, obstetric, and pregnancy-related variables, using independent-samples t-tests, χ^2 tests, Fisher's exact test where cell counts were small, and a logistic regression predicting follow-up completion (Table S8).

1.5 M5. Covariate-adjusted model and clinical cut-offs

The baseline one-way ANOVA on EPDS total scores was complemented by an analysis of covariance including age, education, employment status, marital status, parity, pregnancy planning, mode of conception, and high-risk pregnancy status as covariates, with adjusted marginal means and contrasts against the Low-Risk profile (Table S9).
To convey the clinical meaning of the profiles, the proportion of women scoring at or above the EPDS clinical cut-offs was compared across profiles with χ^2 tests, using both ≥ 9, the screening cut-off validated for the Italian population (Benvenuti et al., 1999), and ≥ 13, the more conservative international threshold for probable depression (Tables S10 and S11). Finally, EPDS scores and profile distribution were compared, unadjusted and for exploratory purposes only, across pregnancy planning, high-risk pregnancy status, parity, and mode of conception (Table S12).

2 Supplementary Results

2.1 Profile labels and sizes

Cluster	Label	n (%)
Cluster 1	Moderately Adjusted	245 (31.6%)
Cluster 2	High-Adversity	113 (14.6%)
Cluster 3	Low-Risk	416 (53.8%)

Note. $N = 774$. Cluster numbering follows the output of the clustering pipeline; profile labels are used throughout the manuscript and in the tables below.

2.2 Item selection, sensitivity analyses, and profile characterisation

Table S0

Candidate Items and Selection for the Cluster Analysis

Instrument	Item	Subscale	M	SD	Variance (raw)	Variance (winsorised)	% extreme	Outcome
MSSS	MSSS_1	Social support	4.06	0.97	0.945	0.823	0.1	Retained

Instrument	Item	Subscale	M	SD	Variance (raw)	Variance (winsorised)	% extreme	Outcome
MSSS	MSSS_2	Social support	4.58	0.64	0.409	0.345	0.3	Retained
MSSS	MSSS_3	Social support	4.50	0.72	0.514	0.387	0.4	Retained
MSSS	MSSS_4	Social support	3.45	1.47	2.175	2.006	2.8	Retained
MSSS	MSSS_5	Social support	3.56	1.90	3.608	3.608	5.2	Retained
MSSS	MSSS_6	Social support	4.64	0.63	0.392	0.215	0.4	Retained
CTQ-SF	CTQ_3	Emotional abuse	1.23	0.68	0.462	0.298	87.3	Retained
CTQ-SF	CTQ_8	Emotional abuse	1.13	0.54	0.295	0.071	92.4	Retained
CTQ-SF	CTQ_14	Emotional abuse	1.36	0.82	0.676	0.448	80.4	Retained
CTQ-SF	CTQ_18	Emotional abuse	1.15	0.60	0.358	0.069	92.5	Retained
CTQ-SF	CTQ_25	Emotional abuse	1.26	0.71	0.507	0.320	85.3	Retained
CTQ-SF	CTQ_9	Physical abuse	1.04	0.35	0.122	0.000	98.2	**Excluded**
CTQ-SF	CTQ_11	Physical abuse	1.07	0.41	0.170	0.000	96.9	**Excluded**
CTQ-SF	CTQ_12	Physical abuse	1.07	0.40	0.158	0.000	95.7	**Excluded**
CTQ-SF	CTQ_15	Physical abuse	1.09	0.46	0.211	0.000	95.5	**Excluded**
CTQ-SF	CTQ_17	Physical abuse	1.04	0.31	0.094	0.000	98.2	**Excluded**
CTQ-SF	CTQ_20	Sexual abuse	1.04	0.33	0.108	0.000	97.8	**Excluded**
CTQ-SF	CTQ_21	Sexual abuse	1.02	0.25	0.060	0.000	99.2	**Excluded**
CTQ-SF	CTQ_23	Sexual abuse	1.04	0.32	0.101	0.000	97.9	**Excluded**
CTQ-SF	CTQ_24	Sexual abuse	1.03	0.26	0.069	0.000	98.4	**Excluded**
CTQ-SF	CTQ_27	Sexual abuse	1.04	0.30	0.092	0.000	98.2	**Excluded**
CTQ-SF	CTQ_5	Emotional neglect	1.51	0.86	0.734	0.520	67.4	Retained
CTQ-SF	CTQ_7	Emotional neglect	1.30	0.67	0.453	0.334	79.2	Retained
CTQ-SF	CTQ_13	Emotional neglect	1.47	0.91	0.831	0.480	72.5	Retained

Instrument	Item	Subscale	M	SD	Variance (raw)	Variance (winsorised)	% extreme	Outcome
CTQ-SF	CTQ_19	Emotional neglect	1.57	0.93	0.858	0.589	63.7	Retained
CTQ-SF	CTQ_28	Emotional neglect	1.36	0.74	0.546	0.408	76.1	Retained
CTQ-SF	CTQ_1	Physical neglect	1.19	0.77	0.588	0.216	93.3	Retained
CTQ-SF	CTQ_2	Physical neglect	1.24	0.70	0.487	0.296	85.9	Retained
CTQ-SF	CTQ_4	Physical neglect	1.03	0.29	0.086	0.000	99.0	**Excluded**
CTQ-SF	CTQ_6	Physical neglect	1.03	0.30	0.090	0.000	99.0	**Excluded**
CTQ-SF	CTQ_26	Physical neglect	1.21	0.67	0.446	0.116	86.7	Retained
CTQ-SF	CTQ_10	Minimization/Denial	3.12	1.62	2.637	2.637	27.8	Retained
CTQ-SF	CTQ_16	Minimization/Denial	4.17	1.01	1.020	0.882	2.6	Retained
CTQ-SF	CTQ_22	Minimization/Denial	4.34	0.92	0.856	0.580	1.9	Retained
MAAS	MAAS_2	Antenatal attachment	4.51	0.73	0.540	0.443	0.6	Retained
MAAS	MAAS_4	Antenatal attachment	4.36	0.90	0.815	0.556	1.9	Retained
MAAS	MAAS_8	Antenatal attachment	3.26	1.17	1.362	1.362	6.1	Retained
MAAS	MAAS_11	Antenatal attachment	4.70	0.60	0.359	0.342	0.4	Retained
MAAS	MAAS_13	Antenatal attachment	4.80	0.52	0.266	0.136	0.3	Retained
MAAS	MAAS_14	Antenatal attachment	4.31	0.83	0.685	0.505	0.9	Retained
MAAS	MAAS_17	Antenatal attachment	2.31	1.12	1.249	1.249	24.5	Retained
MAAS	MAAS_19	Antenatal attachment	4.96	0.34	0.114	0.000	99.5	**Excluded**
MAAS	MAAS_C_1	Antenatal attachment	2.68	1.21	1.475	1.475	14.5	Retained
MAAS	MAAS_C_3	Antenatal attachment	4.63	0.72	0.515	0.416	0.8	Retained
MAAS	MAAS_C_5	Antenatal attachment	4.29	1.00	0.997	0.874	2.2	Retained
MAAS	MAAS_C_6	Antenatal attachment	4.58	0.81	0.652	0.479	0.5	Retained
MAAS	MAAS_C_7	Antenatal attachment	4.59	0.68	0.468	0.368	0.4	Retained

Instrument	Item	Subscale	M	SD	Variance (raw)	Variance (winsorised)	% extreme	Outcome
MAAS	MAAS_C_9	Antenatal attachment	4.78	0.50	0.251	0.152	0.3	Retained
MAAS	MAAS_C_10	Antenatal attachment	3.86	1.03	1.069	0.885	3.9	Retained
MAAS	MAAS_C_12	Antenatal attachment	4.96	0.25	0.061	0.000	99.9	**Excluded**
MAAS	MAAS_C_15	Antenatal attachment	4.96	0.25	0.060	0.000	99.9	**Excluded**
MAAS	MAAS_C_16	Antenatal attachment	4.94	0.33	0.107	0.000	99.9	**Excluded**
MAAS	MAAS_C_18	Antenatal attachment	4.91	0.40	0.158	0.063	0.4	Retained
DAS-7	DAS_1	Dyadic adjustment	3.86	0.96	0.916	0.699	1.3	Retained
DAS-7	DAS_2	Dyadic adjustment	4.24	0.89	0.798	0.490	1.0	Retained
DAS-7	DAS_3	Dyadic adjustment	4.21	0.87	0.762	0.538	0.5	Retained
DAS-7	DAS_4	Dyadic adjustment	4.12	1.09	1.196	0.891	1.6	Retained
DAS-7	DAS_5	Dyadic adjustment	4.29	0.95	0.910	0.735	0.8	Retained
DAS-7	DAS_6	Dyadic adjustment	3.95	1.29	1.652	1.428	3.2	Retained
DAS-7	DAS_7	Dyadic adjustment	4.78	1.38	1.904	1.419	3.0	Retained

Note. N = 774. Items were excluded when their variance fell to zero after winsorisation at the 5th/95th percentiles. "% extreme" is the percentage of responses at the scale minimum (CTQ-SF, MSSS, DAS-7 items) or maximum (excluded MAAS items): the excluded CTQ-SF items show extreme floor effects and the excluded MAAS items extreme ceiling effects. The three CTQ-SF Minimization/Denial items (10, 16, 22), although retained by the variance criterion, were removed in the sensitivity analysis reported in Table S2. Item numbering follows the original instruments. MAAS items labelled with the letter C in the analytic dataset are the negatively worded items reverse-scored per Condon's original scoring; after recoding, all MAAS items run in the same direction (higher = stronger attachment), but the reverse-scored items are more compressed at the scale ceiling (70.8% of responses at the maximum, against 56.3% for the remaining items), which should be borne in mind when item sets are compared.

Table S1

Domain-Level and Instrument-Balanced Sensitivity Analyses

Panel A: Agreement with the item-level solution

Index	Value	Item-level solution
Adjusted Rand index	.757	—
Concordantly classified participants	91.2%	—
Profile sizes (Low-Risk / Mod. Adjusted / High-Adversity)	417 / 254 / 103	416 / 245 / 113
Within-profile concordance — Low-Risk	94%	—
Within-profile concordance — Moderately Adjusted	89%	—
Within-profile concordance — High-Adversity	84%	—
Instrument-balanced solution — Adjusted Rand index	0.728	—
Instrument-balanced solution — Concordantly classified participants	90.4%	—

Index	Value	Item-level solution
Instrument-balanced solution — Profile sizes	421 / 243 / 110	416 / 245 / 113

Panel B: Standardized domain means by profile (domain-level solution)

Domain	Low-Risk	Moderately Adjusted	High-Adversity	Weight
CTQ-SF Emotional abuse	-0.35	-0.18	1.85	1/6
CTQ-SF Emotional neglect	-0.47	-0.02	1.96	1/6
CTQ-SF Physical neglect	-0.30	-0.15	1.58	1/6
DAS-7 (dyadic adjustment)	0.58	-0.78	-0.44	1/6
MSSS (social support)	0.12	-0.05	-0.38	1/6
MAAS (antenatal attachment)	0.54	-0.72	-0.40	1/6

Note. Six standardized domain scores (CTQ-SF emotional abuse, emotional neglect, physical neglect — computed on the retained items; DAS-7, MSSS, and MAAS totals) entered a k-means solution (k = 3) with equal weight. Domain scores were computed from the winsorised, standardized item pool used for the primary analysis. The three-profile solution was substantially reproduced, indicating that the profiles are not driven by the larger number of CTQ-SF items.

Table S2

Sensitivity Analysis Excluding the CTQ-SF Minimization/Denial Items

Index	Value	Item-level solution
Clustering variables	41 items	44 items
Adjusted Rand index (mean across 10 random starts)	.87 (.86–.88)	—
Domain-level solution: ARI excluding M/D domain	.76	—
Domain-level solution: ARI including M/D domain	.69	—
Concordantly classified participants	95.1%	—
Profile sizes (Low-Risk / Mod. Adjusted / High-Adversity)	430 / 237 / 107	416 / 245 / 113
Within-profile concordance — Low-Risk	99%	—
Within-profile concordance — Moderately Adjusted	92%	—
Within-profile concordance — High-Adversity	92%	—

Note. The clustering was repeated after removing CTQ-SF items 10, 16, and 22 (Minimization/Denial scale), which constitute a validity index rather than a measure of maltreatment exposure. The three-profile structure was essentially unchanged. Two analyses are reported: re-clustering at item level on the remaining 41 items, and the domain-level solution of Table S1 run with and without a Minimization/Denial domain, agreement with the primary solution is higher when these items are excluded. Items 16 and 22 correlate at $r = -.99$ and -1.00 with the profile pattern of the clinical CTQ-SF items, behaving as inverse indicators of the same construct; item 10 was the only clustering variable without significant between-profile separation.

Table S3

MSSS (Social Support) — Item-Level Profile Characterisation

Item	Low-Risk	Moderately Adjusted	High-Adversity	Separation (SD units)
MSSS 1	0.19	-0.16	-0.36	0.55
MSSS 2	0.24	-0.09	-0.70	0.94
MSSS 3	0.24	-0.36	-0.10	0.60
MSSS 4	-0.40	0.49	0.41	0.89
MSSS 5	-0.13	0.15	0.16	0.29
MSSS 6	0.18	-0.22	-0.21	0.40

Note. Values are standardized means (z-scores on the winsorised item pool, N = 774) by profile. “Separation” is the range of the three profile means, reported as a descriptive index. Because these items were used to derive the profiles, no inferential tests are performed. MSSS items 4 and 5 are worded as indicators of partner conflict and control and therefore run in the opposite direction to the remaining items, on which higher scores indicate greater perceived

support; because sign reversal leaves Euclidean distances unchanged, item direction does not affect the cluster solution. Item-level means are reported on the original scoring.

Table S4

CTQ-SF (Childhood Maltreatment) — Item-Level Profile Characterisation

Item	Low-Risk	Moderately Adjusted	High-Adversity	Separation (SD units)
CTQ 1	-0.07	-0.02	0.30	0.37
CTQ 2	-0.29	-0.16	1.40	1.69
CTQ 3	-0.24	-0.14	1.19	1.43
CTQ 5	-0.41	0.17	1.14	1.55
CTQ 7	-0.41	-0.15	1.83	2.24
CTQ 8	-0.21	-0.19	1.18	1.39
CTQ 10	0.06	-0.06	-0.09	0.15
CTQ 13	-0.43	-0.02	1.62	2.05
CTQ 14	-0.37	-0.08	1.55	1.92
CTQ 16	0.43	-0.08	-1.43	1.86
CTQ 18	-0.23	-0.16	1.19	1.42
CTQ 19	-0.45	0.08	1.49	1.94
CTQ 22	0.44	-0.03	-1.53	1.97
CTQ 25	-0.34	-0.18	1.66	2.00
CTQ 26	-0.29	0.06	0.91	1.20
CTQ 28	-0.45	-0.11	1.89	2.34

Note. Values are standardized means (z-scores on the winsorised item pool, N = 774) by profile. "Separation" is the range of the three profile means, reported as a descriptive index. Because these items were used to derive the profiles, no inferential tests are performed.

Table S5

MAAS (Antenatal Attachment) — Item-Level Profile Characterisation

Item	Low-Risk	Moderately Adjusted	High-Adversity	Separation (SD units)
MAAS 2	0.30	-0.39	-0.25	0.69
MAAS 4	0.18	-0.25	-0.14	0.43
MAAS 8	0.25	-0.37	-0.13	0.62
MAAS 11	0.34	-0.31	-0.56	0.90
MAAS 13	0.36	-0.43	-0.40	0.79
MAAS 14	0.33	-0.38	-0.38	0.71
MAAS 17	0.12	-0.16	-0.10	0.28
MAAS C 1	-0.05	-0.04	0.27	0.32
MAAS C 3	0.37	-0.48	-0.34	0.85
MAAS C 5	0.21	-0.26	-0.20	0.47
MAAS C 6	0.08	-0.28	0.32	0.60
MAAS C 7	0.15	-0.33	0.18	0.51
MAAS C 9	0.33	-0.34	-0.47	0.80
MAAS C 10	0.20	-0.32	-0.05	0.52
MAAS C 18	0.17	-0.22	-0.16	0.39

Note. Values are standardized means (z-scores on the winsorised item pool, N = 774) by profile. "Separation" is the range of the three profile means, reported as a descriptive index. Because these items were used to derive the profiles, no inferential tests are performed. Items labelled with the letter C are negatively worded and reverse-scored per

Condon's original scoring; after recoding all items run in the same direction, but these items are more compressed at the scale ceiling, so comparisons between item sets defined by this labelling would be confounded with item wording. The three items on which the High-Adversity profile exceeds the Low-Risk profile are C 1 (preoccupation with the baby), C 6 (personification of the fetus as a real person), and C 7 (sense that the baby depends on the mother); in raw scale points the corresponding differences are +0.39, +0.19, and +0.03.

Table S6

DAS-7 (Dyadic Adjustment) — Item-Level Profile Characterisation

Item	Low-Risk	Moderately Adjusted	High-Adversity	Separation (SD units)
DAS 1	0.36	-0.53	-0.16	0.89
DAS 2	0.45	-0.65	-0.24	1.10
DAS 3	0.36	-0.47	-0.31	0.83
DAS 4	0.34	-0.47	-0.21	0.81
DAS 5	0.30	-0.39	-0.27	0.69
DAS 6	0.33	-0.47	-0.19	0.80
DAS 7	0.42	-0.54	-0.36	0.96

Note. Values are standardized means (z-scores on the winsorised item pool, N = 774) by profile. "Separation" is the range of the three profile means, reported as a descriptive index. Because these items were used to derive the profiles, no inferential tests are performed.

Table S7

CTQ-SF Subscale Scores and Proportions Above the Clinical Cut-Off by Profile

Panel A: Subscale scores, M (SD)

Subscale	Low-Risk	Moderately Adjusted	High-Adversity	F	η2
Emotional abuse	5.29 (0.92)	5.61 (1.17)	10.37 (3.92)	—	—
Emotional neglect	5.63 (1.33)	7.00 (1.96)	13.46 (3.67)	—	—
Physical neglect	5.37 (1.09)	5.57 (1.19)	7.22 (2.38)	—	—
Physical abusea	5.01 (0.20)	5.14 (0.54)	6.74 (3.94)	58.20	.131
Sexual abusea	5.03 (0.35)	5.12 (0.89)	5.79 (3.16)	14.86	.037

Panel B: Percentage at or above the moderate-to-severe cut-off

Subscale (cut-off)	Low-Risk	Moderately Adjusted	High-Adversity	χ2	p
Emotional abuse (≥ 13)	0.0% (0/416)	0.0% (0/245)	25.7% (29/113)	176.24	< .001
Emotional neglect (≥ 15)	0.0% (0/416)	0.0% (0/245)	33.6% (38/113)	233.76	< .001
Physical neglect (≥ 10)	0.2% (1/416)	1.2% (3/245)	14.2% (16/113)	71.02	< .001
Physical abuse (≥ 10)	0.0% (0/416)	0.0% (0/245)	12.4% (14/113)	83.40	< .001
Sexual abuse (≥ 8)	0.5% (2/416)	1.6% (4/245)	8.0% (9/113)	26.36	< .001

Note. N = 774. Cut-offs are the moderate-to-severe thresholds (Bernstein & Fink, 1998). a The Physical Abuse and Sexual Abuse subscales contributed no items to the cluster solution and therefore constitute external validators; inferential statistics (*df* = 2, 771) are reported for these two subscales only. Both show pronounced floor effects, so the proportions in Panel B provide the more robust index: with the median at the scale minimum in every profile, the between-group *F* is numerically identical to Levene's test of variance homogeneity (58.20 and 14.86 respectively, verified on the raw data) and therefore does not index a difference in location. The remaining subscales contain cluster-formation items and are reported descriptively.

Table S8

Comparison of Completers and Non-Completers on Baseline Variables

Panel A: Continuous variables, M (SD)

Variable	Completers (n = 200)	Non-completers (n = 574)	Test
Age, years	32.91 (5.33)	32.56 (5.47)	t = 0.80, p = .424, d = 0.07

Variable	Completers (n = 200)	Non-completers (n = 574)	Test
EPDS T0	5.67 (4.72)	5.76 (5.03)	t = −0.22, p = .825, d = −0.02
EPDS T0, within Low-Risk	3.79 (3.50)	4.13 (4.10)	p = .440, d = −0.09
EPDS T0, within Moderately Adjusted	6.81 (4.11)	7.62 (5.31)	p = .287, d = −0.16
EPDS T0, within High-Adversity	8.97 (5.98)	7.97 (5.43)	p = .370, d = 0.18
Gestational week	35.23 (4.47)	35.90 (5.01)	t = −1.66, p = .097, d = −0.14
Years of relationship	9.96 (5.16)	9.92 (5.20)	t = 0.08, p = .934, d = 0.01

Panel B: Categorical variables

Variable	Completers (%)	Non-completers (%)	Test
Antenatal classes attended, %	58.0	40.6	$\chi^2(1) = 18.15$, p < .001, V = .15
Education: university or higher, %	56.0	44.4	$\chi^2(1) = 7.97$, p = .005, V = .10
Married or cohabiting, %	94.5	88.0	$\chi^2(1) = 6.81$, p = .009, V = .09
Primiparous, %	67.0	58.7	$\chi^2(1) = 4.28$, p = .039, V = .07
Previous spontaneous abortion, %	16.5	23.3	$\chi^2(1) = 4.11$, p = .043, V = .07
Third trimester, %	96.5	94.8	$\chi^2(2) = 2.05$, p = .359, V = .05
Stable employment, %	52.0	49.5	$\chi^2(1) = 0.38$, p = .539, V = .02
Planned pregnancy, %	70.5	73.5	$\chi^2(1) = 0.68$, p = .409, V = .03
Natural conception, %	93.5	94.3	$\chi^2(1) = 0.15$, p = .699, V = .01
High-risk pregnancy, %	23.0	27.5	$\chi^2(1) = 1.57$, p = .211, V = .04
Previous high-risk pregnancy, %	3.0	4.7	Fisher, p = .416
Previous elective termination, %	5.0	4.9	Fisher, p = 1.000
Previous stillbirth, %	0.0	0.3	Fisher, p = 1.000

Panel C: Logistic regression predicting follow-up completion (*n* = 755)

Predictor	OR	95% CI	p
Moderately Adjusted (vs. Low-Risk)	0.86	[0.57, 1.30]	.483
High-Adversity (vs. Low-Risk)	1.55	[0.95, 2.53]	.081
Baseline EPDS	0.99	[0.96, 1.03]	.686
Education: university or higher	1.16	[0.80, 1.68]	.441
Married or cohabiting	2.00	[1.02, 3.92]	.045
Primiparous	1.09	[0.75, 1.58]	.668
Gestational week	0.97	[0.94, 1.00]	.057
Antenatal-class attendance	1.85	[1.25, 2.74]	.002

Note. Retention by profile: Low-Risk 103/416 (24.8%), Moderately Adjusted 58/245 (23.7%), High-Adversity 39/113 (34.5%); $\chi^2(2) = 5.29$, *p* = .071, Cramér's V = .08. All comparisons are computed on the full baseline sample (200 completers vs 574 non-completers) using the same antenatal dataset as Table 1 and Table S9. All categorical variables in Panel B have complete data (N = 774). Three continuous variables are incomplete, age (12 missing), gestational week (19), and years of relationship (10). So, under listwise deletion, the valid n is 200 / 562 for age, 197 / 558 for gestational week, and 198 / 566 for years of relationship; all other rows in Panel A rest on 200 / 574. The logistic regression in Panel C includes gestational week and is therefore fitted on 755 cases, whereas the covariate-adjusted model in Table S9 includes age instead and is fitted on 762; this is the only reason the two sample sizes differ. Attrition was associated with engagement with services and social resources rather than with clinical status: neither

baseline depressive symptoms nor profile membership predicted follow-up completion, whereas antenatal-class attendance and marital status did.

Table S9

Cluster Differences in EPDS Scores at T0 Before and After Adjustment for Covariates

Model	F	df	p	η2
Unadjusted	62.52	2, 771	< .001	.140
Adjusted for covariates	58.15	2, 747	< .001	.135

Adjusted marginal means and contrasts

Cluster profile	Unadjusted M	Adjusted M [95% CI]	b vs Low-Risk	p
Low-Risk	4.05	4.06 [2.90, 5.23]	— (ref.)	—
Moderately Adjusted	7.43	7.40 [6.17, 8.63]	+3.34	< .001
High-Adversity	8.32	8.33 [6.94, 9.73]	+4.27	< .001

Note. n = 762 (12 cases excluded for missing age). Covariates were age, education, employment status, marital status, parity, pregnancy planning, mode of conception, and high-risk pregnancy. Among these, only marital status was independently associated with depressive symptoms (p = .004); all others were non-significant. Both at-risk profiles differed from the Low-Risk profile at p < .001. The adjusted marginal means closely match the unadjusted values, indicating that the elevated symptomatology of the two at-risk profiles is not explained by their sociodemographic or obstetric composition but is specifically associated with their psychosocial configuration. The unadjusted effect was verified against the raw item-level data. EPDS = Edinburgh Postnatal Depression Scale.

2.3 Depressive symptoms by profile

Table S10

Antenatal Depressive Symptoms (EPDS) by Profile at T0

Profile	n	M	SD	Mdn	EPDS ≥ 9, %	EPDS ≥ 13, %
Low-Risk	416	4.05	3.96	3	11.1 (46)	5.0 (21)
Moderately Adjusted	245	7.43	5.05	7	35.9 (88)	15.5 (38)
High-Adversity	113	8.32	5.62	8	44.2 (50)	24.8 (28)

Note. N = 774. One-way ANOVA: $F(2, 771) = 62.52$, $p < .001$, $\eta^2 = .140$. Tukey HSD post-hoc comparisons: Moderately Adjusted > Low-Risk (mean difference = 3.38, 95% CI [2.51, 4.25], p < .001) and High-Adversity > Low-Risk (mean difference = 4.27, 95% CI [3.13, 5.42], p < .001), whereas the two at-risk profiles did not differ from each other (mean difference = 0.89, 95% CI [−0.34, 2.12], p = .205). Proportions above cut-off differed across profiles at both thresholds: ≥ 9, $\chi^2(2) = 83.20$, $p < .001$; ≥ 13, $\chi^2(2) = 41.22$, $p < .001$. Cut-offs: ≥ 9 (screening threshold validated for the Italian population; Benvenuti et al., 1999) and ≥ 13 (probable depression). The covariate-adjusted model is reported in Table S9. All values in this table were re-derived from the raw item-level data (N = 774). EPDS = Edinburgh Postnatal Depression Scale.

Table S11

Longitudinal Association Between Profile Membership and Depressive Symptoms (T0–T1)

Panel A: Repeated-measures ANOVA

Effect	F	df	p	Partial η²	Interpretation
Profile (between-subject)	22.740	2, 197	< .001	.188	Profiles differ significantly in mean EPDS scores
Time (within-subject)	0.379	1, 197	.539	.002	No overall change in EPDS over time
Profile × Time	1.400	2, 197	.249	.014	No differential symptom trajectories across profiles

Panel B: EPDS means by profile across assessment waves

Profile	n	EPDS T0, M (SD)	EPDS T1, M (SD)	Post-hoc comparisons
Low-Risk	103	3.79 (3.50)	4.56 (4.66)	Reference group; persistently lowest symptom burden
Moderately Adjusted	58	6.81 (4.11)	6.64 (4.97)	Higher than Low-Risk at T0 (p < .001) and at T1 (p = .023)

Profile	n	EPDS T0, M (SD)	EPDS T1, M (SD)	Post-hoc comparisons
High-Adversity	39	8.97 (5.98)	8.31 (4.69)	Higher than Low-Risk at T0 and T1 (both p < .001); higher than Moderately Adjusted at T0 (p = .040)

Panel C: Proportion above the EPDS clinical cut-offs at T1

	Low-Risk (n = 103)	Moderately Adjusted (n = 58)	High-Adversity (n = 39)	$\chi^2(2)$	p
EPDS >= 9, % (n)	18.4 (19)	32.8 (19)	46.2 (18)	11.69	.003
EPDS >= 13, % (n)	7.8 (8)	10.3 (6)	15.4 (6)	1.83	.400

Note. Longitudinal subsample: *n* = 200 women with EPDS data at both T0 and T1 (25.8% of the baseline sample). The significant profile effect indicates that profiles identified during pregnancy are associated with differences in depressive symptom levels that persist across the perinatal period; the non-significant Time effect and Profile × Time interaction indicate that symptom levels did not systematically change over time in any profile. The analysis addresses the persistence of between-profile differences in symptoms, not the stability of profile membership, which the design does not allow to be assessed. T0 descriptives for the Low-Risk profile differ slightly from Table S10 (full sample *M* = 4.05, *SD* = 3.96; longitudinal subsample *M* = 3.79, *SD* = 3.50) owing to subsample composition. At the ≥ 13 threshold the T1 comparison is not significant, reflecting the small cell counts in the longitudinal subsample; the ≥ 9 threshold is therefore emphasised in the main text. All three panels rest on the same 200 women; the linkage procedure that identifies them is described in Supplementary Methods M4. EPDS = Edinburgh Postnatal Depression Scale.

Table S12

Exploratory Subgroup Comparisons: EPDS at T0 and Profile Distribution Across Obstetric Subgroups

Subgroup	n (%)	EPDS T0, M (SD)	Test (p)	d	% LR / MA / HA	$\chi^2(2)$ (p)
Planned pregnancy	563 (72.7)	5.52 (4.77)	$t(772) = -1.93$ (.055)	−0.16	55.8 / 31.4 / 12.8	6.25 (.044)
Unplanned pregnancy	211 (27.3)	6.33 (5.37)			48.3 / 32.2 / 19.4	
No high-risk pregnancy	570 (73.6)	5.64 (4.80)	$t(772) = -0.87$ (.384)	−0.07	53.5 / 31.8 / 14.7	0.06 (.972)
High-risk pregnancy	204 (26.4)	6.01 (5.35)			54.4 / 31.4 / 14.2	
Primiparous	471 (60.9)	5.42 (4.45)	$t(772) = -2.12$ (.034)	−0.16	56.5 / 29.7 / 13.8	3.61 (.165)
Multiparous	303 (39.1)	6.23 (5.61)			49.5 / 34.7 / 15.8	
Natural conception	728 (94.1)	5.80 (5.02)	$t(772) = 1.76$ (.084)	+0.20	53.2 / 31.7 / 15.1	3.00 (.224)
Medically assisted reproduction	46 (5.9)	4.83 (3.53)			63.0 / 30.4 / 6.5	

Note. N = 774. Exploratory, unadjusted comparisons; the adjusted model that addresses the same substantive question is reported in Table S9. Welch's *t*-test for continuous comparisons; Cramér's *V* was .09, .01, .07, and .06 for the four χ^2 tests respectively. Profile distribution differed across subgroups only by pregnancy planning, the High-Adversity profile being more frequent among unplanned pregnancies. Given the small size of the medically assisted reproduction subgroup (*n* = 46) and the resulting cell counts, that comparison should be interpreted with caution. High-risk pregnancy was recorded in the study database as a single binary indicator, without a breakdown by clinical condition; it is the same variable reported in Table 1 of the main text. LR = Low-Risk; MA = Moderately Adjusted; HA = High-Adversity; EPDS = Edinburgh Postnatal Depression Scale.

3. References cited in this supplement

- Benvenuti, P., Ferrara, M., Niccolai, C., Valoriani, V., & Cox, J. L. (1999). The Edinburgh Postnatal Depression Scale: Validation for an Italian sample. *Journal of Affective Disorders, 53*(2), 137–141. https://doi.org/10.1016/S0165-0327(98)00102-5
- Bernstein, D. P., & Fink, L. (1998). *Childhood Trauma Questionnaire: A retrospective self-report manual*. The Psychological Corporation.
- Condon, J. T. (1993). The assessment of antenatal emotional attachment: Development of a questionnaire instrument. *British Journal of Medical Psychology, 66*(2), 167–183. https://doi.org/10.1111/j.2044-8341.1993.tb01739.x
- Hennig, C. (2007). Cluster-wise assessment of cluster stability. *Computational Statistics & Data Analysis, 52*(1), 258–271. https://doi.org/10.1016/j.csda.2006.11.025
